# Normalization of non‐canonical Wnt signalings does not compromise blood‐brain barrier protection conferred by upregulating endothelial Wnt/β‐catenin signaling following ischemic stroke

**DOI:** 10.1111/cns.13661

**Published:** 2021-05-31

**Authors:** Ya‐bin Ji, Tian‐xi Wang, Qiang Gao, Xiao‐wen Huang, Junlei Chang

**Affiliations:** ^1^ Shenzhen Key Laboratory of Biomimetic Materials and Cellular Immunomodulation Institute of Biomedicine and Biotechnology Shenzhen Institute of Advanced Technology Chinese Academy of Sciences Shenzhen China; ^2^ Department of Neurology Nanfang Hospital Southern Medical University Guangzhou China; ^3^ Baiyun affiliated Cerebrovascular Hospital Nanfang Hospital Baiyun Branch Southern Medical University Guangzhou China; ^4^ Department of Neurosurgery The First Affiliated Hospital of Zhengzhou University Zhengzhou University Zhengzhou China

**Keywords:** blood‐brain barrier, endothelial cell, ischemic stroke, Wnt signaling, β‐catenin

## Abstract

**Background:**

Endothelial canonical (Wnt/β‐catenin) and non‐canonical Wnt signalings (Wnt/PCP and Wnt/Ca^2+^) promote blood‐brain barrier (BBB) development and antagonize each other. However, the effects of ischemic stroke on endothelial canonical and non‐canonical Wnt signalings are unclear. Further, how non‐canonical Wnt signalings are influenced by upregulation of endothelial Wnt/β‐catenin signaling and subsequently affect BBB function following ischemic stroke have not been studied.

**Methods:**

First, we determined the levels of Wnt signaling markers including TCF/LEF1 transcription activity, *Axin2* mRNA, phospho‐JNK^Thr183/Tyr185^, and NFAT in brain endothelial cells (ECs) with the deletion of Wnt receptor *Frizzled (Fzd)*4 or *Fzd6*, the two most abundant Fzds in brain ECs. Next, we observed the effect of ischemia/reperfusion injury on Wnt signalings in brain ECs and adult mice. Last, we assessed the changes of non‐canonical Wnt signalings and BBB injury in the early stage of ischemic stroke in mice with endothelial β‐catenin activation (β‐cat mice).

**Results:**

*Fzd4* or *Fzd6* deletion dampened both Wnt/β‐catenin and Wnt/PCP signalings but enhanced Wnt/Ca^2+^ signaling in brain ECs. Both canonical and non‐canonical Wnt signalings in brain ECs were downregulated after ischemia/reperfusion injury *in vitro* and *in vivo*. Upregulating endothelial Wnt/β‐catenin signaling in β‐cat mice normalized the downregulated non‐canonical Wnt signalings, which did not compromise its protective effects on BBB integrity and endothelial tight junction following ischemic stroke.

**Conclusions:**

The BBB protection induced by upregulation of endothelial Wnt/β‐catenin signaling may be not interfered by the normalization of non‐canonical Wnt signalings in the early stage of ischemic stroke.

## Graphical abstract

The BBB protection induced by upregulation of Wnt/β‐catenin signaling may be not interfered by the normalization of non‐canonical Wnt signalings in the early stage of ischemic stroke.

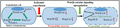

## 1 INTRODUCTION

The blood‐brain barrier (BBB) is comprised of endothelial cells (ECs), pericytes, basement membrane, and astrocyte, among which, the barrier function is mainly attributed to the ECs and regulated by endothelial Wnt/β‐catenin signaling (Wnt/β‐cat),^1^, ^2^ also named as canonical Wnt signaling. The canonical Wnt signaling is involved in many embryonic development processes, and abnormal canonical Wnt signaling causes various diseases, including ischemic stroke, neurodegenerative diseases, and various kinds of cancers.^3^, ^4^, ^5^ In addition, as shown by plenty of evidences, Wnt proteins can also activate signaling pathways independent of β‐catenin, mainly including Wnt/planar cell polarity (PCP) and Wnt/Ca^2+^ signaling, which are together called non‐canonical Wnt signalings.^4^ The Wnt/β‐cat signaling is involved in cell differentiation and proliferation; Wnt/PCP signaling regulates cytoskeleton and cell polarization; Wnt/Ca^2+^ signaling is associated with inflammation and neurodegeneration.^4^ Previous studies have shown that Wnt/β‐cat and Wnt/PCP signalings in ECs both played crucial roles to control the integrity of ECs and the barrier function of BBB,^1^, ^2^, ^6^ and Wnt/Ca^2+^ signaling is involved in the EC growth and migration,^7^ indicating synergistic effects can be induced by different types of Wnt signaling. Interestingly, nevertheless, some clues have hinted the existence of antagonism between canonical and non‐canonical Wnt signalings.^8^, ^9^

Our previous study showed that after ischemic stroke, the BBB integrity was disrupted in mice with conditional deletion of endothelial *Gpr124*, a Wnt7‐specific coactivator of Wnt/β‐cat signaling.^10^ The disruption of BBB function was fully rescued by genetic activation of endothelial β‐catenin,^10^ suggesting that the BBB breakdown in the early stage of ischemic stroke could be treated by manipulating endothelial Wnt/β‐cat signaling. Besides, some other studies including ours showed that the treatment with glycogen synthase kinase 3β (GSK3β) inhibitors (IM‐12, TWS119, and lithium) significantly improved the outcomes of mice with ischemic stroke by upregulating Wnt/β‐cat signaling.^11^, ^12^, ^13^ Since upregulating endothelial Wnt/β‐cat signaling has been considered as a novel therapeutic method for protecting BBB after ischemic stroke, and the antagonisms between canonical and non‐canonical Wnt signalings have been suggested to exist,^8^, ^9^ it is necessary to determine the changes of non‐canonical Wnt signalings and their consequences while manipulating endothelial Wnt/β‐cat signaling in the treatment of ischemic stroke.

Here, we aimed to investigate the interplay of canonical and non‐canonical Wnt signalings in brain ECs under normal state or ischemic stroke in vivo and in vitro, and examine the changes of non‐canonical Wnt signalings during the upregulation of endothelial Wnt/β‐cat signaling by employing a genetic mouse model with endothelial β‐catenin activation.

## 2 MATERIALS AND METHODS

### 2.1 Cell culture and OGD/R treatment

The mouse brain endothelial cell line bEnd.3 was purchased from American Type Culture Collection (CRL‐2299) and cultured in Dulbecco modified eagle medium (DMEM) containing 10% fetal bovine serum (FBS) and 1% penicillin/streptomycin. The *Frizzled (Fzd) 4* and *Fzd6* knockout cell lines were constructed using CRISPR‐Cas9 basing on the bEnd.3 cell line by GENEWIZ company (Beijing, China). Both knockout cell lines had been authenticated by DNA sequencing (Supplementary Figure S1). Primary brain ECs were obtained from brains of 8‐ to 10‐week‐old adult C57BL/6 wild‐type mice which were minced and disaggregated. After centrifugation at 400 *g* for 5 min, cell pellets were resuspended in EGM‐2MV medium (LONZA) with 10% FBS and 4 μg/ml puromycin and plated into fibronectin‐precoated plates (10 μg/ml for 30 min at 37°C). 3 days later, medium was discarded and cells were washed with PBS twice and medium was replaced with EGM‐2MV/10% FBS. All cultures were maintained in a humidified 5% CO_2_ incubator at 37°C and routinely passaged when 80%–90% confluent. The optimal durations of Wnt5a for activating Wnt/PCP and Wnt/Ca^2+^ signalings by measuring the relative levels of phospho‐JNK^Thr183/Tyr185^ and nuclear factor of activated T cells (NFAT) protein in bEnd.3 cell line had been determined in preliminary experiments (Supplementary Figure S2).

The cells were exposed to oxygen‐glucose deprivation (OGD). Briefly, cultured medium was replaced by Dulbecco's Modified of Eagle's Medium (Solarbio) and the cultured cells were put in a Modular Incubator Chamber (Billups‐Rothenberg) with 0.5%–1% O_2_ and 99% N_2_, which was monitored with an O_2_ analyzer (HNZA). We assessed the effect of different durations of oxygen‐glucose recovery (OGR) after 6 hour (h)‐OGD for cell vitality and 3 h‐OGR was chosen in the following experiments (Supplementary Figure S3). Hence, after 6 h‐OGD, cells were returned to normal culture conditions for 3 h‐OGR.

### 2.2 Real‐time quantitative PCR

Total RNA was extracted using the Direct‐zol RNA MiniPrep kit (Zymo Research). RNA was reverse transcribed using iScript Reverse Transcription Supermix for real‐time quantitative PCR (RT‐qPCR) according to the manufacturer's instructions (Vazyme). RT‐qPCR was carried out on a StepOnePlus Real‐Time PCR System using the Power SYBR Green method (Roche). RNA expression was calculated using the comparative Ct method normalized to Actin. Data were expressed relative to a calibrator using the 2^−(ΔΔCt)^ method. Primer sequences of all genes are shown in Supplementary Table S1.

### 2.3 Dual luciferase assay

For a typical dual luciferase assay, wild type (WT), *Fzd4* ^−/−^, and *Fzd6* ^−/−^ bEnd.3 cells were transfected by lentivirus with TCF/LEF (cells: viral particles = 1:5; QIAGEN) or NFAT firefly luciferase (cells: viral particles = 1:5; QIAGEN), and Renilla control (cells: viral particles = 1:2.5; QIAGEN) in 96‐well plates. Four replicate wells were prepared for each group. After 48 h, cells were stimulated with Wnt3a (R&D Systems) for 24 h, or Wnt5a (R&D Systems) for 30 min. Firefly and Renilla luciferase activities were measured using the Dual Stop and Glo system (Promega). Reporter activity was calculated as Firefly/Renilla activity in each well.

### 2.4 Animal protocol

Male 8‐ to 10‐week‐old C57BL/6 mice were obtained from the Beijing Vital River Laboratory Animal Technologies Co. Ltd. To induce endothelial β‐catenin (encoded by *Ctnnb1*) constitutive activation, *Cdh5‐CreER* mice were crossed with mice bearing the *Ctnnb1^lox(ex3)^* allele to generate *Ctnnb1^lox(ex3)/+^*; *Cdh5‐CreER* mice (termed β‐cat), and *Ctnnb1^+/+^*; *Cdh5*‐*CreER* control mice (termed WT). The recombination efficiency of *Ctnnb1* gene in brain ECs of β‐cat mice has been confirmed in our previous study^10^ and preliminary experiments. In RNA‐seq of brain ECs, we found due to only exon3 being deleted while other exons were expressed normally, *Ctnnb1* mRNA expression in β‐cat mice did not apparently decrease compared with WT mice, but the Wnt/β‐cat target genes *Axin2, Apcdd1*, and *Nkd1* significantly increased (data not shown). Meanwhile, non‐canonical Wnt signaling target genes *Pfn2, Vangl2, Celsr3, DAAM1, CamkII, and NFATc1/c3* expression in brain ECs of β‐cat mice had no significant changes (Supplementary Figure S4). 8‐ to 10‐week‐old mice were treated with tamoxifen (2 mg/10 g body weight) in corn oil through an oral feeding needle every other day for 5 days with a total of three doses per mouse. Mice were allowed to recover from tamoxifen treatment‐related toxicities (undergo washout) for 8 days before undergoing any other surgical procedures or experiments.

Animals were housed individually in the animal facility. All procedures performed on mice were approved and carried out in accordance with the Animal Care and Use Committee of Shenzhen Institute of Advanced Technology, Chinese Academy of Sciences. Randomized animals were used in the sham operation and ischemia/reperfusion (I/R) group. For the surgical procedures, anesthesia was induced with 4% isoflurane in an induction chamber; anesthesia was maintained with 2% isoflurane delivered through a face mask (RWD). A heating pad was used to maintain each mouse's core temperature at 37 ± 0.5°C throughout the surgical procedure. A modified intraluminal filament model was used to induce transient middle cerebral artery occlusion (MCAO) as previously described.^14^ After 60 min of MCAO, reperfusion was established by retracting the filament. Animals had free access to food and water throughout the 24 h reperfusion period. Neurological deficit scores were evaluated at 24 h post‐MCAO by a blind observer according to the following scoring system: 0 = normal extension of both forelimbs; 1 = adduction of affected forelimb; 2 = the grip strength of affected forelimb decreased significantly; 3 = leaning to the opposite side; 4 = circling to the contralateral side; and 5 = no autonomic activity.

### 2.5 Infarct size analysis

The infarct region in sections with H & E staining was defined as the area with reduced staining or areas containing eosinophilic‐necrotic cell bodies. The boundaries between regions of infarct and adjacent normal brain were clearly delineated. Tissue sections were photographed using a ZEISS microscope (Axio Imager 2 Pol), and the infarct area was measured using ImageJ software (National Institutes of Health, USA). To eliminate the contribution of postischemic edema to the area of injury, the infarct area (%) was calculated as follows: [(area of the non‐ischemic hemisphere – non‐infarct area of ischemic hemisphere)/area of the non‐ischemic hemisphere] × 100%.

### 2.6 Western blot analyses

For the analyses of marker proteins of canonical and non‐canonical Wnt signaling or BBB components, ECs or ischemic hemisphere tissues of mice were obtained and analyzed by Western blot. The proteins of ECs or cerebral tissues were isolated and harvested by RIPA lysis buffer (Solarbio) supplemented with protease and phosphatase inhibitors and quantified by BCA assay according to the standard protocols. Equal amounts of protein lysates from each sample were fractionated on 10% SDS‐PAGE gels. The proteins were transferred onto polyvinylidene fluoride membranes. The membranes were then incubated with primary antibodies including rabbit anti‐phospho‐JNK^Thr183/Tyr185^ (CST), rabbit anti‐JNK (CST), rabbit anti‐active β‐catenin (CST), mouse anti‐NFAT (Santa Cruz), rabbit anti‐phospho‐CamkII^Thr286^ (CST), rabbit anti‐CamkII (CST), rabbit anti‐Laminin (Sigma‐Aldrich), rabbit anti‐zonula occludens‐1 (ZO‐1) (Thermal Fisher), rabbit anti‐Desmin (Abcam), at a 1:1000 dilution. β‐actin (1:3000; CST, MA) or GAPDH (1:3000; CST) served as the loading control for normalization. The nucleus NFAT is the marker of Wnt/Ca^2+^ signaling, so nucleus fraction was obtained using Nuclear and Cytoplasmic Protein Extraction Kit (TransGen biotech) according to the manufacturer's protocols, while PCNA (1:1000; CST) was used as the normalization control in nucleus protein analysis. The membranes were incubated with the respective secondary antibodies and blots were developed and imaged using the GelView 6000M system (BioLight).

### 2.7 Immunofluorescence staining

Frozen 7‐μm‐thickness sections were allowed to dry on Adhesion Microscope slides (CITOTEST) at room temperature before being rehydrated in PBS. Sections were blocked in 10% normal goat serum (Thermal Fisher) in PBS +0.2% Triton X‐100 for 1 h at room temperature. Samples were incubated at 4°C with the following antibodies in PBS +5% goat serum +0.2% Triton X‐100: hamster anti‐mouse CD31 (1:200; Millipore), rabbit anti‐ZO‐1 (1:100; Thermal Fisher), rabbit anti‐Claudin‐5 (1:40; Thermal Fisher), rabbit anti‐Laminin (1:100; Sigma‐Aldrich), rabbit anti‐Desmin (1:100; Abcam), and donkey anti‐mouse IgG (1:200, Jackson ImmunoResearch). Excess antibody was removed by rinsing in PBS for 5 min, 3 times. Samples were then incubated at room temperature for 1 h with the Cy3 goat anti‐hamster IgG (Jackson ImmunoResearch), Alexa Fluor 488 donkey anti‐rabbit IgG (Jackson ImmunoResearch) diluted 1:500 in PBS +5% BSA +0.2% Triton X‐100 for 1 h at room temperature. Excess antibody was removed by rinsing in PBS for 5 min, 3 times. Slides were mounted in antifading mounting medium with DAPI (Solarbio) and imaged with an Olympus microscope to obtain 20× or 40× images. The whole‐brain image was taken under low‐power microscope and adjusting white balance through healthy hemisphere. The optical density (OD) value was measured by an experimenter blinded to group allocation with ImageJ. Immunofluorescence signal area or density was quantified and normalized by CD31 signal area in 5 to 8 random ischemic areas of cortex. Pericyte coverage was quantified by measuring the staining signal length of pericytes or ECs with ImageJ. All tests were performed by an experimenter who was unaware of the identity of experimentations.

### 2.8 FACS sorting of brain ECs

Brains of adult mice were minced and disaggregated in Type IV collagenase (400 U/ml, Worthington), Dispase (1.2 U/ml, Worthington), and DNase I (32 U/ml, Worthington) in PBS with Ca^2+^ and Mg^2+^ (GIBCO) at 37°C for 60 min, with gentle trituration every 10 min. After that, the disaggregated cells were washed with cold PBS and filtered through a 40‐μm mesh. After centrifugation, cell pellets were resuspended in 10 ml 20% BSA and centrifuged at 1000 *g* at 4°C for 25 min to remove the myelin. The pellets were then resuspended in PBS/3% FBS/0.1% NaN3/2 mM EDTA and blocked rat anti‐mouse CD16/32 (Mouse BD Fc Block, BD Pharmingen) for 5 min on ice and labeled with antibodies according to our previous study^10^ for 1 h at 4°C. The stained cells were sorted into EGM‐2/MV medium (Clontech) with an Aria II sorter (BD) or analyzed with an LSRII analyzer (BD) at Fluorescence‐activated cell sorting (FACS) Facility, and FACS data were analyzed using FlowJo software (TreeStar).

### 2.9 RNA‐seq

For mouse brain ECs, the total RNA was extracted using the Arcturus picopure RNA Isolation Kit (Applied Biosystems). According to our previous study,^10^ we performed quantitative 3′ end SEQ. The sequencing was performed on Illumina‐hiseq‐2500 platform (Illumina) in single‐ended 50 bp format. TopHat was used to map the original sequence reading to the mouse genome (mm10), and customized R scripts were used to calculate the frequency of Refseq genes. Then, the original counts were standardized by using the pruning average (TMM) method of M value, and compared with Bioconductor software package "edgeR." Readings per gigabase per million (RPKM) are also calculated from raw counts. In at least one sample, if RPKM ≥1, fold change ≥1.5, *p* ≤ 0.05, the differentially expressed genes can be identified. Gene richness was analyzed by David database.

### 2.10 Statistical analysis

Statistical analysis was performed with the SPSS 16.0 for Windows software package (SPSS). Data were expressed as means ± standard error (SE). One‐way analysis of variance (ANOVA) was used to determine statistical diﬀerences in all of the observed indicators from the diﬀerent groups. The least significant difference (LSD) *t* test or the Dunnett T3 test, if the variance was heterogeneous, was used to further analyze differences. Two groups were analyzed by unpaired *t* tests. Statistical significance was defined as *p* < 0.05.

## 3 RESULTS

### 3.1 The effect of *Fzd4* or *Fzd6* deletion on canonical and non‐canonical Wnt signalings in brain endothelial cells

The Fzd proteins are well known as the essential membrane receptors of Wnt ligands and mediate their downstream signalings.^3^ Our data showed that both *Fzd4* and *Fzd6* were abundantly expressed with their mRNA levels much higher than any of the other eight Fzd genes in bEnd.3 cell line and primary brain ECs of adult mice (Supplementary Figure S5A,B). The growth rates of *Fzd4* or *Fzd6* deletion cells were lower than that of wild‐type cells (Supplementary Figure S5C), indicating Fzd4 and Fzd6 played important roles in brain ECs.

**FIGURE 1:**
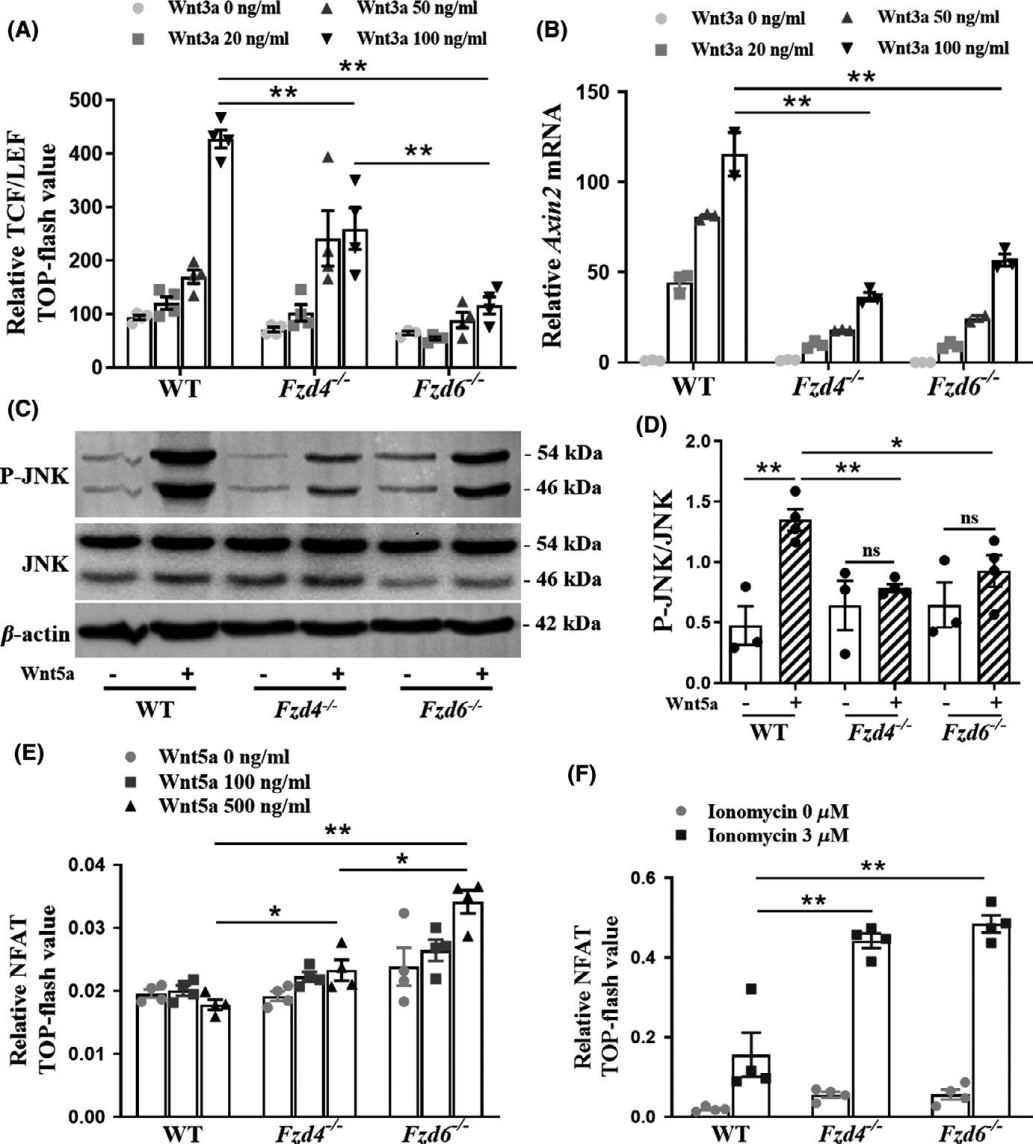
The effect of *Fzd4* or *Fzd6* deletion on canonical and non‐canonical Wnt signalings in brain endothelial cells. (A) The relative OD value of TCF/LEF in TOP‐flash test in WT, *Fzd4* ^−/−^, *Fzd6* ^−/−^ bEnd.3 cells with 0 to 100 ng/ml Wnt3a protein treatment for 24 h. *n* = 4 biological replicates per condition. (B) Expression of *Axin2* mRNA in WT, *Fzd4* ^−/−^, *Fzd6* ^−/−^ bEnd.3 cells, with 0 to 100 ng/ml Wnt3a protein treatment for 24 h, as assessed by RT‐qPCR. *n* = 3 biological replicates per condition. (C) The P‐JNK, JNK protein levels in WT, *Fzd4* ^−/−^, *Fzd6* ^−/−^ bEnd.3 cells with 200 ng/ml Wnt5a protein treatment for 30 min. (D) The ratio of P‐JNK and JNK from the protein bands in (C). *n* = 3–4 samples per group. (E, F) The relative OD value of NFAT in TOP‐flash test in WT, *Fzd4* ^−/−^, *Fzd6* ^−/−^ bEnd.3 cells with 0, 100, or 500 ng/ml Wnt5a protein treatment for 60 min or ionomycin with 0 or 3 µM for 24 h. *n* = 4 biological replicates per condition. Data are mean ± SE, *p < 0.05, **p < 0.01. One‐Way ANOVA followed by LSD *t* test. Fzd, frizzled; WT, wild type; P‐JNK, phospho‐JNK^Thr183/Tyr185^; NFAT, nuclear factor of activated T cells; ns, not significant

A plethora of evidences have suggested that Fzd4 serves as the principal receptor for Wnt/β‐cat signaling in brain ECs,^15^ while the role of Fzd6 in this signaling is yet unclear. As shown in Figure 1A and B, deleting *Fzd4* or *Fzd6* resulted in significant decrease of TCF/LEF TOP‐flash value (100 ng/ml Wnt3a treatment: *F* = 35.128, *p* = 0.001; *p* < 0.0001) and *Axin2* mRNA level (100 ng/ml Wnt3a treatment: *F* = 50.088, *p* < 0.0001; *p* = 0.001), two of the indicators of Wnt/β‐cat signaling activity, and the TCF/LEF TOP‐flash value in *Fzd6* ^−/−^ cells was lower than that in *Fzd4* ^−/−^ cells (*p* = 0.004), in a Wnt3a dose‐dependent manner, indicating that Fzd6, in addition to Fzd4, could also play a necessary role in mediating Wnt/β‐cat signaling.

Then, we detected the phosphorylation of JNK, the key downstream process in Wnt/PCP signaling.^16^ We found that with Wnt5a stimulation, the ratio of phospho‐JNK^Thr183/Tyr185^ (P‐JNK) to JNK was significantly reduced in *Fzd4* ^−/−^ and *Fzd6* ^−/−^cells, compared with WT cells (*F* = 9.888, *p* = 0.0053; *p* = 0.0263), and Wnt5a failed to upregulate P‐JNK in *Fzd4* ^−/−^ and *Fzd6* ^−/−^cells (*t* = 0.8296, *p* = 0.4446; *t* = 1.281, *p* = 0.2563; Figure 1C,D). These data here supported that both Fzd4 and Fzd6 had the certain function in Wnt/PCP signaling.

Next, NFAT, the main transcription factor of Wnt/Ca^2+^ signaling,^17^ was investigated. Results showed that with Wnt5a stimulation at 500 ng/ml, or ionomycin stimulation at 3 µM, the NFAT TOP‐flash value in *Fzd4* ^−/−^ and *Fzd6* ^−/−^ cells was higher than those in WT cells (Wnt5a stimulation: *F* = 31.195, *p* = 0.0290, *p* = 0.0001; ionomycin stimulation: *F* = 24.867, *p* < 0.0001, *p* < 0.0001; Figure 1E,F), indicating that Wnt signalings mediated by Fzd4 or Fzd6 might be against Wnt/Ca^2+^ signaling.

Taken together, these data provided the following evidences: first, in addition to Fzd4, Fzd6 might be another key membrane receptor for Wnt/β‐cat signaling; second, although the role and importance might be different, Fzd4 and Fzd6 mediated both Wnt/PCP and Wnt/β‐cat signalings; third, the antagonism might exist between Wnt/Ca^2+^ signaling and other Wnt signalings mediated by Fzd4 or Fzd6 (namely Wnt/β‐cat and Wnt/PCP signaling).

### 3.2 Endothelial canonical and non‐canonical Wnt signalings were downregulated after OGD/R

**FIGURE 2:**
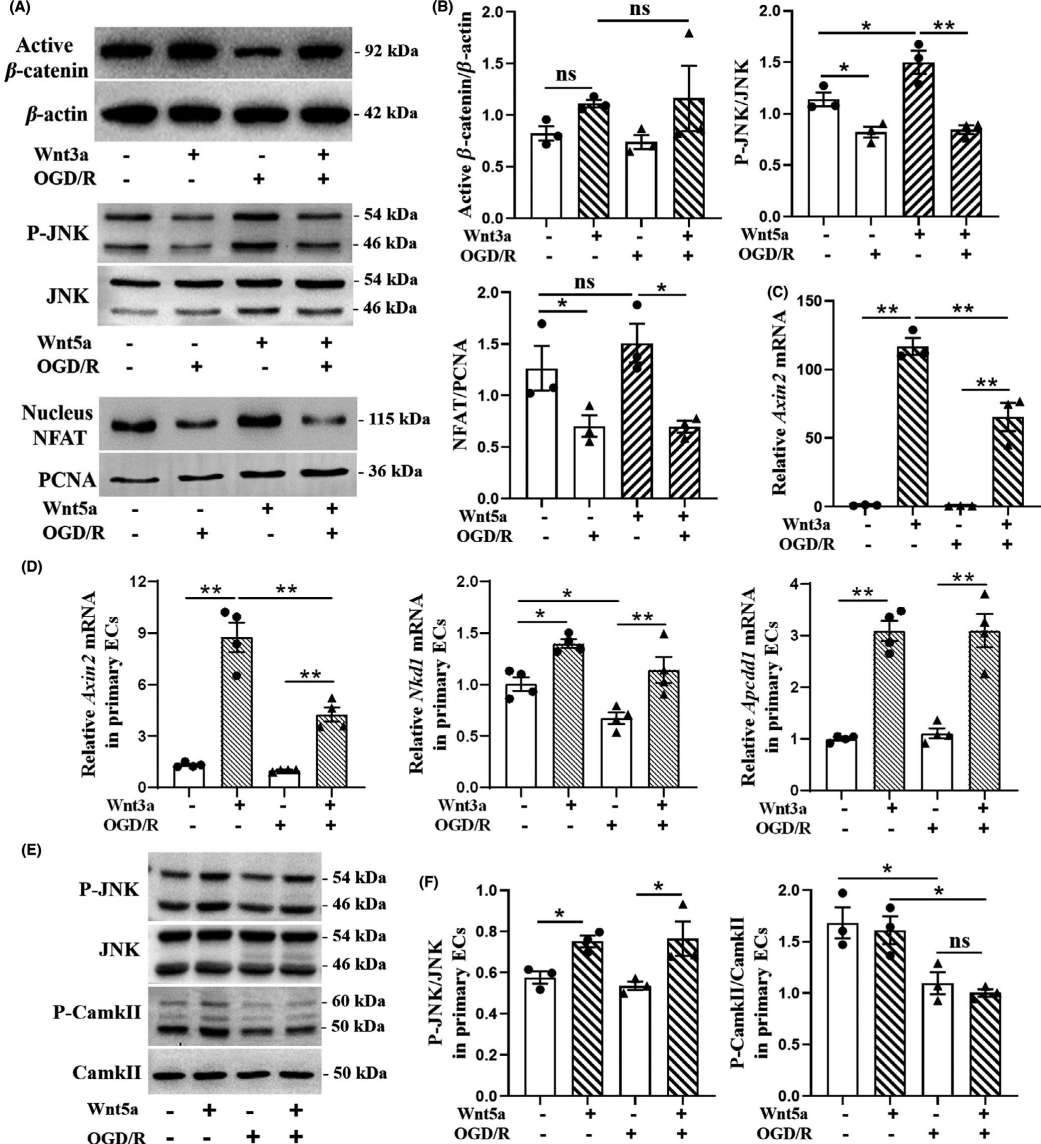
Canonical and non‐canonical Wnt signalings in brain endothelial cells were both downregulated by OGD/R. (A) After OGD/R, the relative active β‐catenin, P‐JNK, and nucleus NFAT protein levels in bEnd.3 cell line with 100 ng/ml Wnt3a for 24 h, 200 ng/ml Wnt5a for 30 min, and 200 ng/ml Wnt5a for 60 min, respectively. (B) Quantitation of the bands in (A). *n* = 3 samples per condition. One‐way ANOVA followed by Dunnett T3 test for active β‐catenin and LSD *t* test for other proteins. (C) Expression of *Axin2* mRNA in bEnd.3 cell line after OGD/R, with 100 ng/ml Wnt3a protein treatment for 24 h, as assessed by RT‐qPCR. n = 3 biological replicates per condition. One‐way ANOVA followed by LSD *t* test. (D) Expression of *Axin2, Nkd1*, and *Apcdd1* mRNA in primary brain ECs of adult mice after OGD/R, with 100 ng/ml Wnt3a protein activation for 24 h, as assessed by RT‐PCR. *n* = 4 biological replicates per condition. One‐way ANOVA followed by LSD *t* test. (E) After OGD/R, P‐JNK, JNK, P‐CamkII, and CamkII protein levels in primary brain ECs were measured by Western blot, with 200 ng/ml Wnt5a protein stimulation for 60 min. (F) The ratios of P‐JNK and JNK, P‐CamkII, and CamkII from the protein bands in (E). *n* = 3 samples per group. One‐way ANOVA followed by LSD *t* test. Data are mean ± SE, *p < 0.05, **p < 0.01. OGD/R, oxygen‐glucose deprivation and recovery; P‐JNK, phospho‐JNK^Thr183/Tyr185^; P‐CamkII, phospho‐CamkII^Thr286^; NFAT, nuclear factor of activated T cells; ECs, endothelial cells; ns, not significant

In order to investigate the effects of OGD/R on the endothelial canonical and non‐canonical Wnt signalings, we evaluated the indicators of these signalings in bEnd.3 cells and primary brain ECs of adult mice. Under normal circumstances, Wnt3a significantly upregulated *Axin2* mRNA level (*F* = 88.81, *p* < 0.0001; Figure 2C) in bEnd.3, and *Axin2* (*F* = 57.24, *p* < 0.0001), *Nkd1* (*F* = 14.60, *p* = 0.0197) and *Apcdd1* (*F* = 36.80, *p* < 0.0001) in primary brain ECs (Figure 2D); after OGD/R, in cells with Wnt3a stimulation, the *Axin2* mRNA level in bEnd.3 (*F* = 88.81, *p* = 0.0013) and in primary brain ECs (*F* = 57.24, *p* = 0.0001) was still significantly lower than cells in normal control (Figure 2C,D), suggesting that Wnt/β‐cat signaling activity in ECs was downregulated by OGD/R. The reason of no significant differences of active β‐catenin protein in different conditions may be due to the low sensitivity of Western blot tests (Figure 2A,B). Relative P‐JNK protein level was significantly upregulated in bEnd.3 cells (*F* = 18.33, *p* = 0.0359; Figure 2A,B) and in primary brain ECs (*F* = 6.093, *p* = 0.0139; Figure 2E,F) with Wnt5a stimulation, while it was just a slight trend of increase in nucleus NFAT protein expression (*F* = 6.904, *p* = 0.6947; Figure 2A,B). After OGD/R, apparent decreases of P‐JNK and nucleus NFAT protein were observed in bEnd.3 cells and phospho‐CamkII^Thr286^ (P‐CamkII) in primary brain ECs with Wnt5a stimulation (*F* = 18.33, *p* = 0.0011; *F* = 6.904, *p* = 0.0253; *F* = 9.114, *p* = 0.0240; Figure 2A,B,E,F). The decrease of P‐JNK in primary brain ECs after OGD/R was not apparent with or without Wnt5a stimulation, but the mRNA levels of two markers of Wnt/PCP signaling *Pfn2* (*F* = 7.328, *p* = 0.0252) and *Vangl2* (*F* = 36.04, *p* = 0.0001) significantly decreased after OGD/R (Supplementary Figure S6A). These results indicated that after OGD/R, both canonical and non‐canonical Wnt signalings were downregulated.

### 3.3 Upregulating endothelial Wnt/β‐cat signaling after I/R resulted in the normalization of downregulated non‐canonical Wnt signalings

**FIGURE 3:**
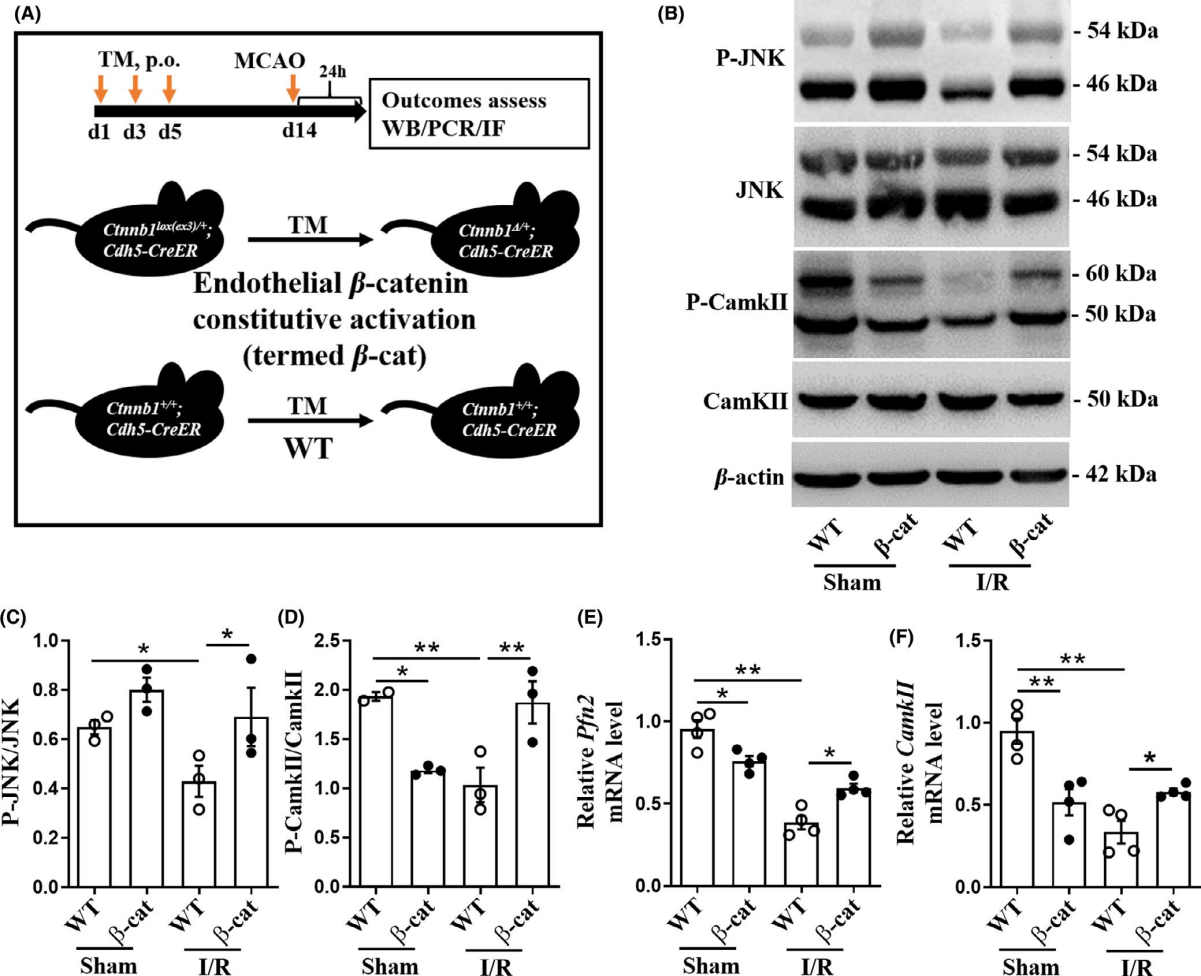
The downregulated non‐canonical Wnt signalings in mouse brain following I/R were normalized by endothelial‐specific β‐catenin activation. (A) Breeding schema for endothelial cell conditional *Ctnnb1* exon3 deletion by crossing to *Cdh5‐CreER* mice, deleting exon3, and converting the *Ctnnb1^lox(ex3)^* allele into a *Ctnnb1^ex3Δ^* allele. (B) After I/R, P‐JNK, JNK, P‐CamkII, and CamkII protein levels in brains of mice were measured by Western blot. (C, D) The ratios of P‐JNK and JNK, P‐CamkII, and CamkII from the bands in (B). *n* = 3 mice per group. (E, F) Expression of *Pfn2* and *CamkII*, as assessed by RT‐qPCR, in ischemic hemisphere of mice. *n* = 4 mice per group. Data are mean ± SE, *p < 0.05, **p < 0.01. One‐way ANOVA followed by LSD *t* test. TM, tamoxifen; MCAO, middle cerebral artery occlusion; WT, wild type; I/R, ischemia/reperfusion; P‐JNK, phospho‐JNK^Thr183/Tyr185^; P‐CamkII, phospho‐CamkII^Thr286^

Given that upregulating endothelial Wnt/β‐cat has been demonstrated as a novel potential treatment for BBB protection following ischemic stroke,^10^, ^11^, ^12^, ^13^ we wondered if the treatment after ischemic stroke would induce any changes of non‐canonical Wnt signalings. The mice with endothelial β‐catenin constitutive activation were generated, shown in Figure 3A. As shown in Figure 3B–F, the relative P‐JNK (*F* = 4.615, *p* = 0.0410) and P‐CamkII (*F* = 8.664, *p* = 0.0070) protein, and the mRNA levels of *Pfn2* (*F* = 37.36, *p* < 0.0001) and *CamkII* (*F* = 15.17, *p* = 0.0001) in WT mice with I/R were apparently reduced, compared with WT mice with sham operation; after I/R, P‐JNK (*p* = 0.0310), P‐CamkII (*p* = 0.005), *Pfn2* (*p* = 0.0127), and *CamkII* (*p* = 0.0231) mRNA levels in β‐cat mice were higher than that in WT mice. In order to confirm whether endothelial non‐canonical Wnt signalings were also normalized after the upregulation of endothelial Wnt/β‐cat signaling, we determined the changes of non‐canonical Wnt signaling markers in primary brain ECs with Wnt/β‐cat signaling activated by Wnt3a protein after OGD/R. We found that after Wnt3a upregulated Wnt/β‐cat signaling, only the relative P‐CamkII protein level increased significantly (*F* = 5.687, *p* = 0.0326; Supplementary Figure S6B,C), while *Pfn2*, *Vangl2* mRNA, and relative P‐JNK protein level did not change significantly (Supplementary Figure S6A–C). These data suggest that the upregulation of endothelial Wnt/β‐cat signaling after I/R would synergistically normalize inhibited Wnt/PCP and Wnt/Ca^2+^ signalings in brain tissues, but only Wnt/Ca^2+^ signaling in brain ECs. It is worth mentioning that with sham operation, the *Pfn2* mRNA level was lower in β‐cat mice than that in WT mice (*F* = 37.36, *p* = 0.0207; Figure 3E). Also, for Wnt/Ca^2+^ signaling, the ratio of P‐CamkII to CamkII protein (*F* = 8.664, *p* = 0.015; Figure 3D) and the mRNA level of *CamkII* (*F* = 15.17, *p* = 0.0029; Figure 3F) were lower in β‐cat mice than that in WT mice, supporting that both Wnt/PCP and Wnt/Ca^2+^ signalings in brain tissues were inhibited by upregulating endothelial Wnt/β‐cat signaling.

### 3.4 The normalization of non‐canonical Wnt signalings did not compromise the BBB protective effect of upregulating endothelial Wnt/β‐cat signaling after I/R

**FIGURE 4:**
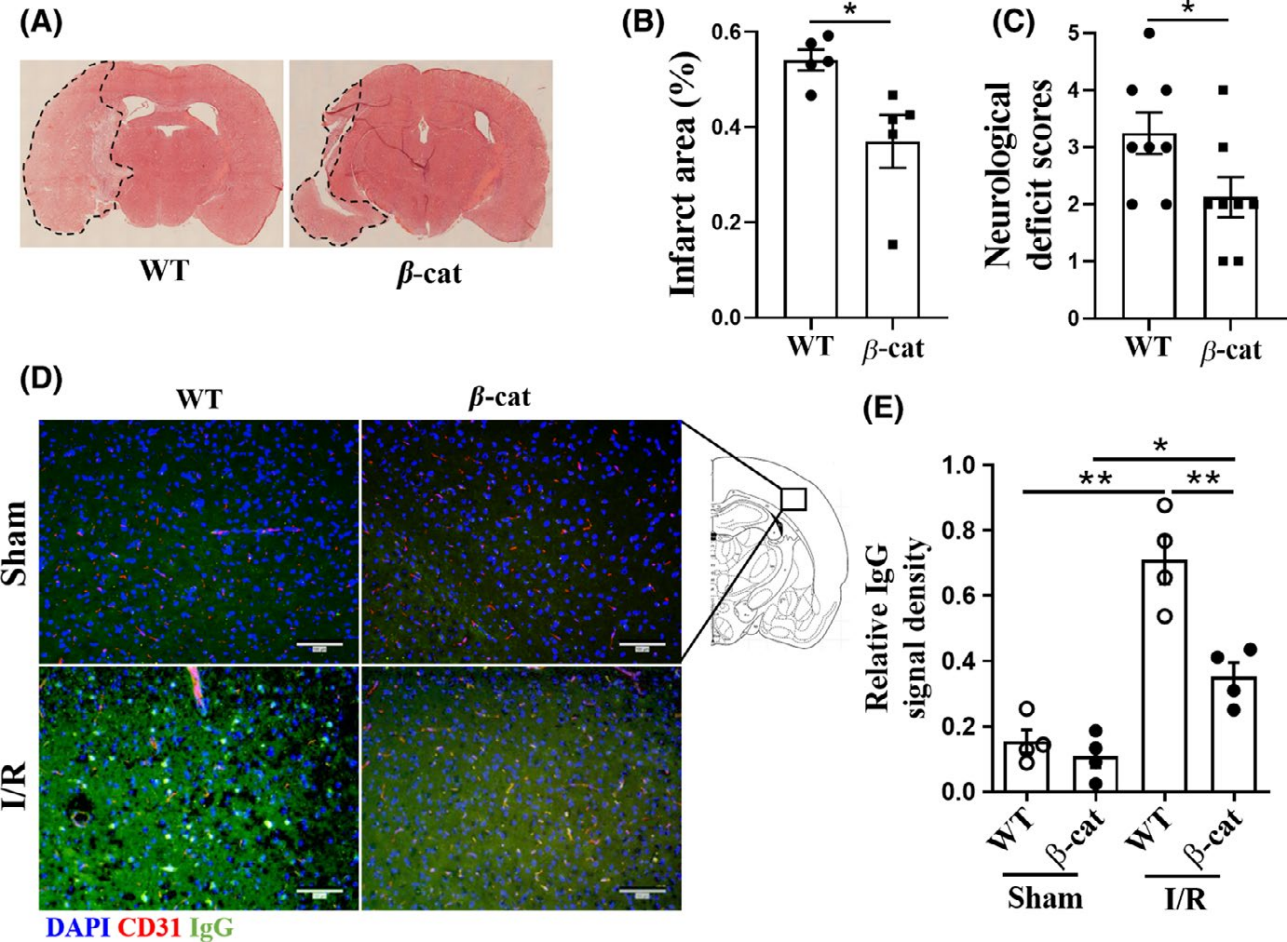
The stroke outcomes and BBB breakdown were improved in endothelial‐specific β‐catenin activation mice after I/R. (A) Photomicrographs showing infarct area in coronal sections of the brains of mice after I/R. (B) Quantification of infarct areas as determined by H & E staining, and encircled by dotted line. n = 5 mice per group. Unpaired *t* test. (C) The neurological deficit scores in WT and β‐cat mice after I/R. *n* = 8 mice per group. Unpaired *t* test. (D) Immunofluorescence staining of leaked endogenous plasma IgG (green) from blood vessels (red) after I/R. Scale bar, 100 µm. (E) Quantification of plasma IgG leakage. *n* = 4 mice per group. One‐way ANOVA followed by LSD *t* test. Data are mean ± SE, *p < 0.05, **p < 0.01. I/R, ischemia/reperfusion; β‐cat, *Ctnnb1^lox(ex3)^* ^/+^; *Cdh5*‐*CreER* mice; WT, *Ctnnb1* ^+/+^; *Cdh5*‐*CreER* mice

To identify downstream effectors of upregulating endothelial Wnt/β‐cat signaling after I/R, the components of BBB, including endothelial tight junctions, basement membrane, and pericyte coverage, were examined. First of all, our data showed that smaller infarct areas were found in β‐cat mice (37.0 ± 5.6%) than that in WT mice (54.1 ± 2.2%; *t* = 2.870, *p* = 0.0208; Figure 4A,B), and the neurological deficit scores in β‐cat mice were also lower than that in WT mice (*t* = 2.220, *p* = 0.0434; Figure 4C). The BBB leakage was assessed by mouse blood IgG extravasation from blood vessels in infarct areas. Our results showed that blood IgG leakage was rescued in β‐cat mice compared with WT mice (*F* = 31.09, *p* = 0.0012; Figure 4D,E), indicating that upregulating endothelial Wnt/β‐cat signaling significantly improved the BBB function after I/R.

**FIGURE 5:**
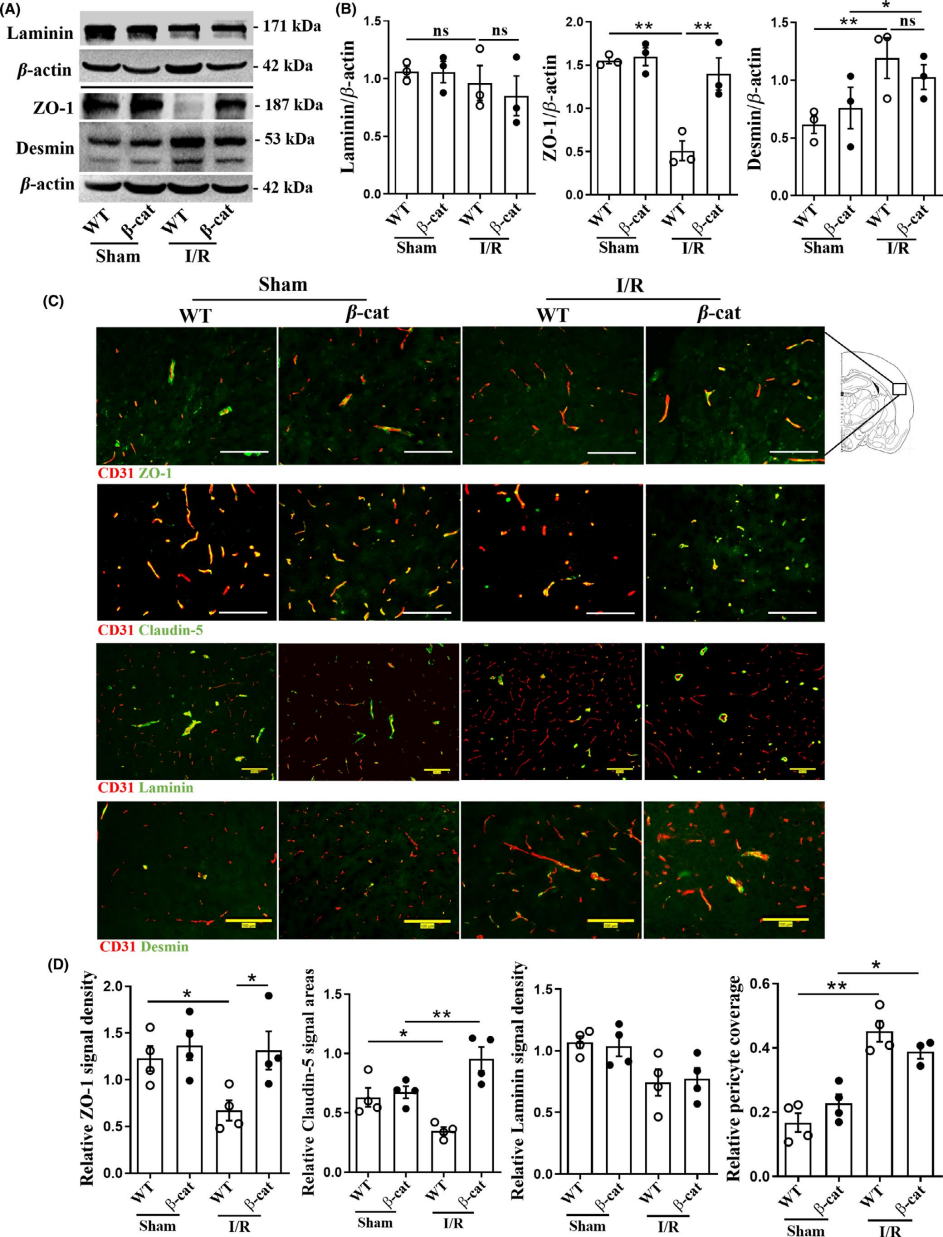
The endothelial tight junction was protected in brains of endothelial‐specific β‐catenin activation mice after I/R. (A) The basement membrane (Laminin), tight junction (ZO‐1), and pericyte (Desmin) marker protein levels in ischemic hemisphere of mice were measured by Western blot. (B) Quantitation of the protein bands in (A). *n* = 3 mice per group. (C) Co‐immunofluorescence staining for ZO‐1, Claudin‐5, Laminin, and Desmin (green) with CD31 (red) in brain infarcted regions. The yellow signals showed positive signals on blood vessels. Scale bar, 100 µm. (D) Relative positive signal densities or areas were normalized to the CD31 signal area. 5 – 8 low‐power fields per mice were randomly selected. n = 4 mice per group. Data are mean ± SE, *p < 0.05, **p < 0.01. One‐way ANOVA followed by LSD *t* test. I/R, ischemia/reperfusion; ZO‐1, zonula occludens‐1; β‐cat, *Ctnnb1^lox(ex3)^* ^/+^; *Cdh5‐CreER* mice; WT, *Ctnnb1* ^+/+^; *Cdh5‐CreER* mice; ns, not significant

Then, the protein levels of the markers of BBB components in ischemic hemisphere of mice were detected by Western blot. As shown in Figure 5A and B, there were no obvious differences in these markers between β‐cat and WT mice with sham operation. After I/R, ZO‐1 protein, one of the main tight junction proteins, significantly decreased in WT mice, but not in β‐cat mice (*F* = 17.54, *p* = 0.0038; Figure 5A,B). Consistently, more ZO‐1 and Claudin‐5 coverage on blood vessels were observed by immunofluorescence staining in the infarct area of β‐cat mice than that of WT mice (*F* = 4.276, *p* = 0.013; *F* = 12.45, *p* = 0.0003; Figure 5C,D). However, for marker proteins of basement membrane and pericyte in Western blot (Laminin; *F* = 0.6242, *p* = 0.9219; Desmin: *F* = 3.316, *p* = 0.8460; Figure 5A,B) and immunofluorescence staining (Laminin: *F* = 4.086, *p* = 0.9954; Desmin: *F* = 21.23, *p* = 0.4973; Figure 5C,D), no significant changes were observed in the brains of β‐cat mice compared with those of WT mice. Taken together, although downregulated non‐canonical Wnt signalings were normalized, the BBB protection induced by upregulating endothelial Wnt/β‐cat signaling following I/R was not weakened.

## 4 DISCUSSION

Interacting with more than 15 receptors and co‐receptors in seven protein families, Wnt proteins‐elicited signalings are complex and involved in extensive physiological and pathological processes in cells.^4^ Due to the hydrophobicity, Wnt proteins are not able to enter across cytomembrane in the initial phase of Wnt signaling. With a large extracellular Cysteine‐rich domain mediating its binding with Wnt proteins, Fzd proteins can help Wnt proteins to transduce their actions into cytoplasm. The complex network of Fzd‐Wnt signalings has been outlined by numerous evidences: (1) Fzd4 is mainly involved in endothelial Wnt/β‐cat signaling,^3^, ^15^, ^18^ while also has a certain function in Wnt/PCP signaling^19^; (2) Fzd6 acts with a central role in Wnt/PCP signaling^3^, ^20^; (3) Fzd2 and Fzd5 may mediate Wnt/Ca^2+^ signaling,^18^ while there is no evidence of the direct association of Wnt/Ca^2+^ signaling with Fzd4 or Fzd6. Even though Wnt signalings have been widely studied, there are still some questions remained to be answered: (1) One previous study showed that the *Fzd4* deletion in ECs failed to completely abolish the physiological function of Wnt/β‐cat signaling,^18^ suggesting that in addition to Fzd4, there might be another Fzd subtype mediating Wnt/β‐cat signaling. We wondered if Fzd6, one of the two most abundant Fzd subtype in brain ECs, also mediates Wnt/β‐cat signaling. (2) Previous studies have shown that Wnt/β‐cat, Wnt/PCP, and Wnt/Ca^2+^ signalings collectively contribute to the endothelial growth, angiogenesis, and BBB formation,^1^, ^2^, ^6^, ^7^ while the antagonisms between canonical (Wnt/β‐cat) and non‐canonical (Wnt/PCP and Wnt/Ca^2+^) signalings in physiological status have been implicated.^8^, ^9^ Given that it has been reported that Wnt/β‐cat signaling augmentation could protect BBB after cerebral I/R,^10^, ^11^, ^12^ if upregulating Wnt/β‐cat signaling could affect Wnt/PCP and Wnt/Ca^2+^ signalings becomes a new question.

It has been shown that Fzd4 could activate Wnt/β‐cat signaling by specifically interacting with Wnt proteins and norrin, an atypical Fzd ligand, to play an essential role in barrier maintenance and plasticity in brain ECs.^15^, ^21^ Our study supported that Fzd4 was not the only Fzd receptor in mediation of endothelial Wnt/β‐cat signaling. We found that TCF/LEF TOP‐flash value and *Axin2* mRNA levels were significantly reduced not only in *Fzd4* ^−/−^ cells, but also in *Fzd6* ^−/−^ cells, indicating that Fzd6 also had certain functions in Wnt/β‐cat signaling. Fzd6 has been shown to regulate Wnt/PCP signaling as its prevalent role,^20^ but there is no evidence of Fzd6 being involved in Wnt/β‐cat signaling in brain ECs until this study. Our results demonstrated the dual function of Fzd6 in mediating canonical and non‐canonical Wnt signalings in brain ECs, providing a foundation for the cooperation between canonical and non‐canonical Wnt signalings during endothelial growth and BBB formation. On the contrary, Golan T and his colleagues found that human Fzd6 could act as a negative regulator of the Wnt/β‐cat signaling by inhibiting the ability of β‐catenin to activate the transcription in human 293 T and cancer cell lines.^22^ This contradiction might be due to different cell types. Another study reported that Fzd6 could activate β‐catenin in patients with nail dysplasia caused by germline Fzd6 mutations,^23^ which is consistent with our results.

Canonical and non‐canonical Wnt signalings have been reported to antagonize each other. By competitively binding Fzd proteins to activate Wnt/PCP and Wnt/Ca^2+^ signaling, Wnt5a could inhibit the interaction between Wnt3a and Fzd proteins to suppress Wnt/β‐cat signaling.^8^ Another previous study showed that CamkII could phosphorylate β‐catenin independently of GSK3β, inducing the inhibition of Wnt/β‐cat signaling.^9^ However, the interplay among different Wnt signalings in pathological state is still unclear. In this study, we investigated the changes of these signalings after I/R, and our data showed that all three endothelial Wnt signalings at protein or gene levels were downregulated after I/R. Even though we believed that the inhibition of all Wnt signalings might not share the same mechanism in different types of pathologies, I/R was considered as the acute and destructive injury to break the physiological antagonisms among these Wnt signalings and downregulate all Wnt signalings. This point of view was verified by our results, suggesting that except for upregulation of Wnt/β‐cat signaling, the recovery of inhibited non‐canonical Wnt signalings to normal levels might also be required for BBB protection after ischemic stroke. In this case, non‐canonical Wnt signalings agonist might be useful for treating ischemic stroke. On the other hand, although some evidences suggested the existence of the antagonism between canonical and non‐canonical Wnt signalings, the interaction between Wnt/PCP and Wnt/Ca^2+^ signaling still remains obscure. Our results showed that NFAT TOP‐flash values of *Fzd4* ^−/−^ and *Fzd6* ^−/−^ cells were significantly higher than those in WT cells. In consideration of the role of Fzd6 and Fzd4 in Wnt/PCP signaling, the result provided a clue of the possible antagonism between Wnt/Ca^2+^ and Wnt/PCP signaling in ECs.

Previous studies showed BBB breakdown occurred rapidly following reperfusion within several hours and reached to a peak around at 24 to 48 h after ischemia.^24^, ^25^, ^26^, ^27^ Furthermore, hemorrhagic transformation in ischemic stroke patients is detected usually within 24 to 36 h following intravenous thrombolysis therapy and thrombectomy. Therefore, in this study we chose to allow the reperfusion to carry on for 24 h in order to fully observe the effect of non‐canonical Wnt signalings normalization for BBB protection induced by upregulation of endothelial Wnt/β‐cat signaling. Our previous study showed that increasing Wnt/β‐cat signaling protected BBB by regulating endothelial tight junctions in the acute stage of ischemic stroke.^10^ Consistently, we also found the protection for tight junctions in mice with endothelial β‐catenin activation in the early stage of ischemic stroke, and BBB protection was not weakened even though antagonistic non‐canonical Wnt signalings were synchronously upregulated, which is the first time to observe the changes of non‐canonical Wnt signalings in the process of BBB protection by manipulating endothelial Wnt/β‐cat signaling. Interestingly, we found that the changes of non‐canonical Wnt signalings in primary brain ECs were not completely consistent with that in brain tissues after upregulating endothelial Wnt/β‐cat signaling, which showed that downregulated Wnt/PCP and Wnt/Ca^2+^ signalings in brain tissues could be normalized, while only Wnt/Ca^2+^ signaling was normalized in primary brain ECs. It must be mentioned that Wnt proteins in brain tissue are secreted principally by neurons and astrocytes,^28^, ^29^ so here we observed the changes of Wnt signalings in the whole‐brain tissues, not only ECs, because the upregulation of endothelial Wnt/β‐cat signaling would affect non‐canonical Wnt signalings not only in ECs, but also in other brain cells. In fact, pericytes, astrocytes, and microglia are also related to BBB integrity through PDGFR‐β, AQP4, Poldip2 or inflammation‐related signaling pathways.^30^, ^31^, ^32^, ^33^ At the same time, Wnt signaling is not the only one to maintain endothelial homeostasis through tight junction protein‐dependent mechanism.^34^, ^35^, ^36^ Even so, our data provide the evidence for Wnt signaling as an important link in maintaining BBB integrity.

In conclusion, our findings demonstrate that: (1) After ischemic stroke, canonical and non‐canonical Wnt signalings were downregulated, while non‐canonical Wnt signalings could be normalized by upregulating endothelial canonical Wnt signaling. However, we did not investigate the effect of targeted manipulation of non‐canonical Wnt signalings on BBB injury after ischemic stroke, which may be worthy of further study. (2) The protection of the BBB by upregulation of Wnt/β‐cat signaling depends on protecting the endothelial tight junction in the early stage of ischemic stroke, which was not disturbed by normalization of non‐canonical Wnt signalings. (3) It is worth mentioning that except Fzd4, Fzd6 also plays an important role in endothelial Wnt/β‐cat signaling. Combined with previous studies,^3^, ^15^, ^18^, ^19^, ^20^ Fzd4 and Fzd6 might have dual mediating roles in both Wnt/β‐cat and Wnt/PCP signalings, which will be beneficial for a more comprehensive understanding of Wnt signalings in brain endothelial cells.

## CONFLICT OF INTEREST

The authors declare that they have no conflict of interest.

## Supporting information

Figure S1

Figure S2

Figure S3

Figure S4

Figure S5

Figure S6

Supinfo

Table S1

## Data Availability

The data that support the findings of this study are available in the supplementary material of this article.
