# Supplementary figures and images for: Normalization of non‐canonical Wnt signalings does not compromise blood‐brain barrier protection conferred by upregulating endothelial Wnt/β‐catenin signaling following ischemic stroke
Source: CNS Neurosci Ther. 2021 May 31;27(9):1085–96. doi: 10.1111/cns.13661 (PMC8339534; doi:10.1111/cns.13661)

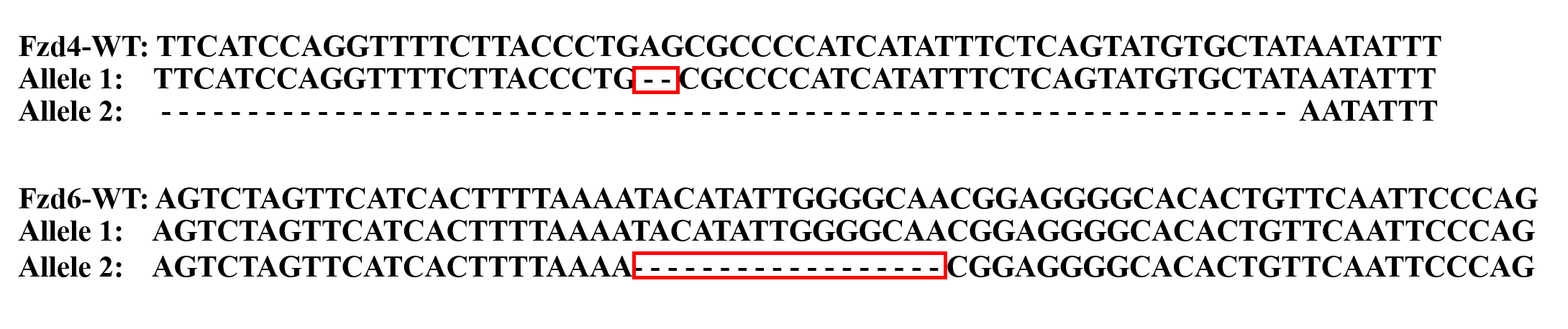

Supplement: Supplementary file 1 — Figure S1 [file CNS-27-1085-s005.tif]

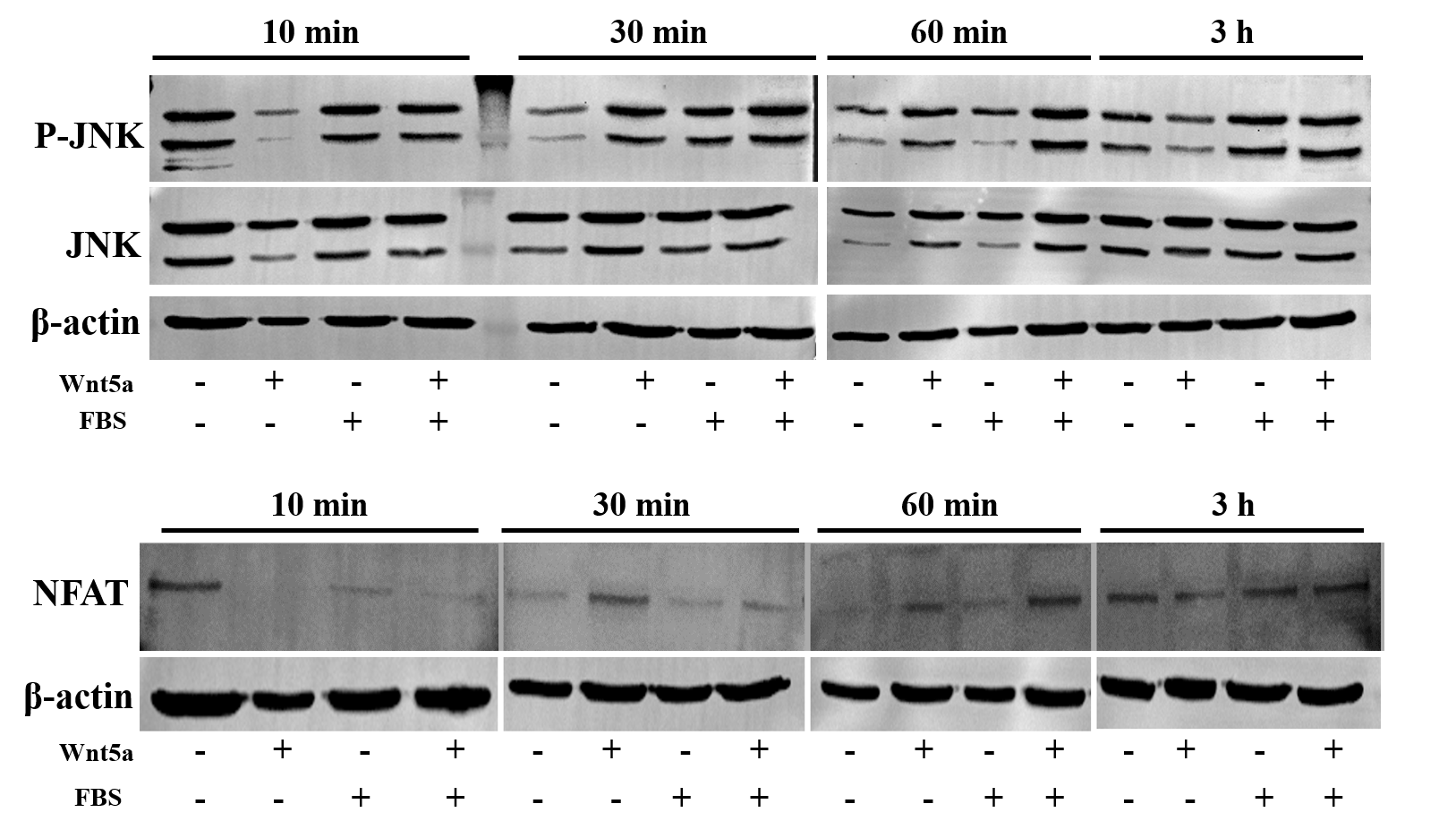

Supplement: Supplementary file 2 — Figure S2 [file CNS-27-1085-s008.tif]

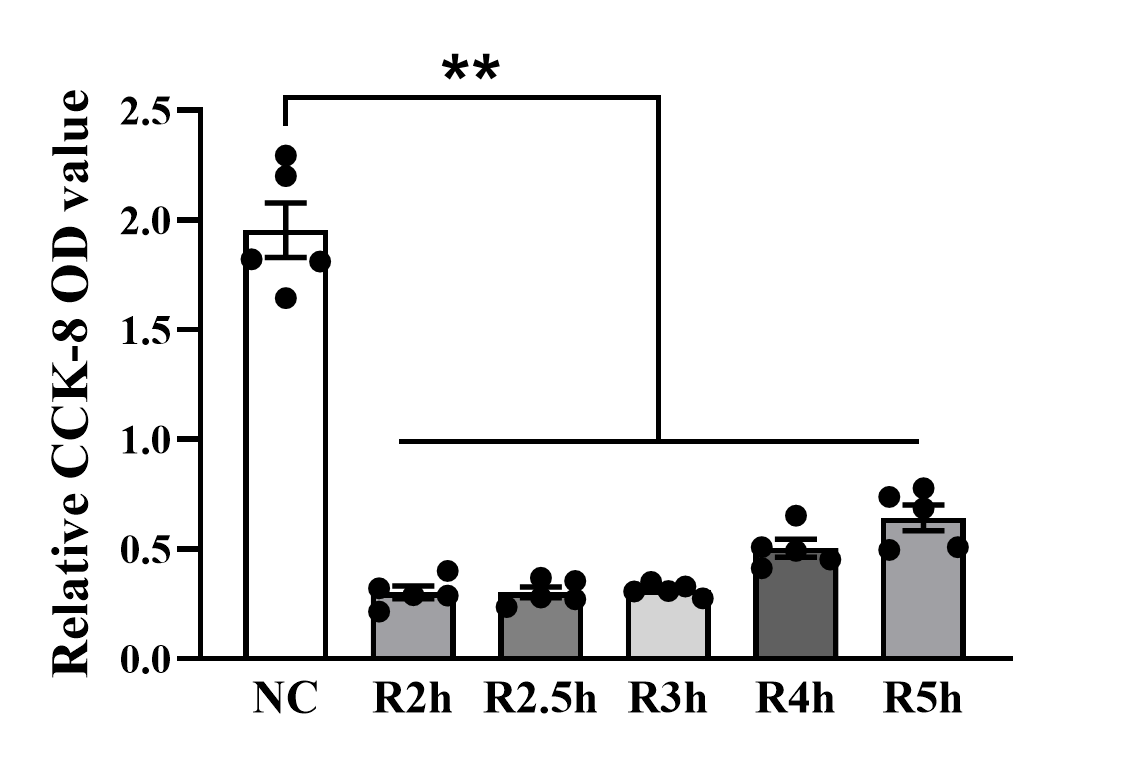

Supplement: Supplementary file 3 — Figure S3 [file CNS-27-1085-s004.tif]

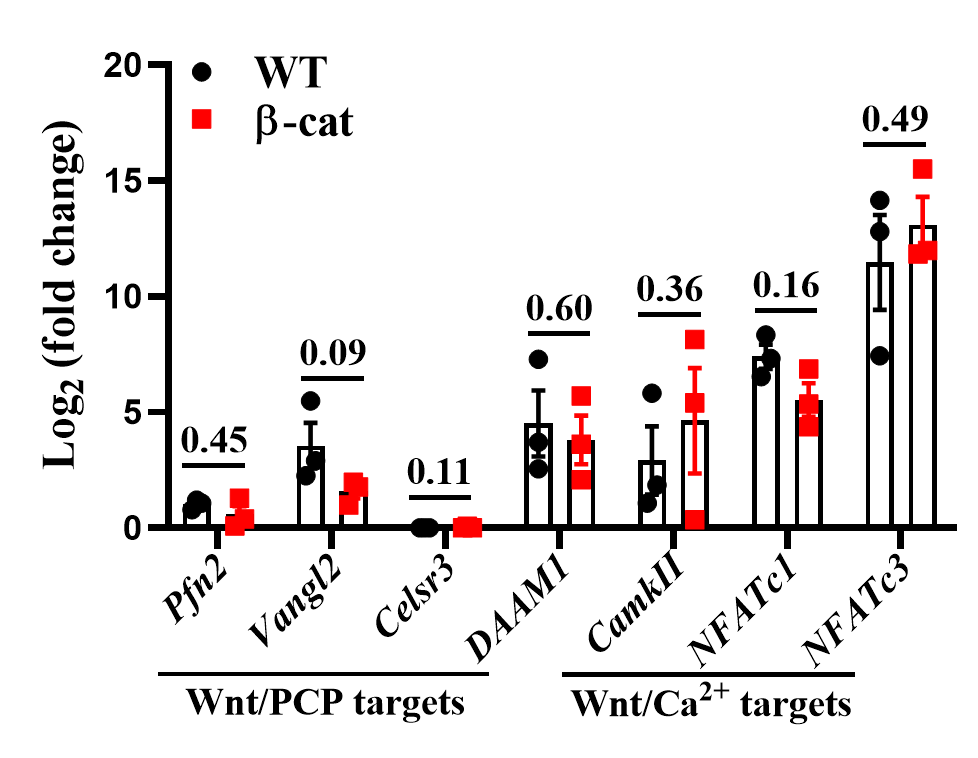

Supplement: Supplementary file 4 — Figure S4 [file CNS-27-1085-s001.tif]

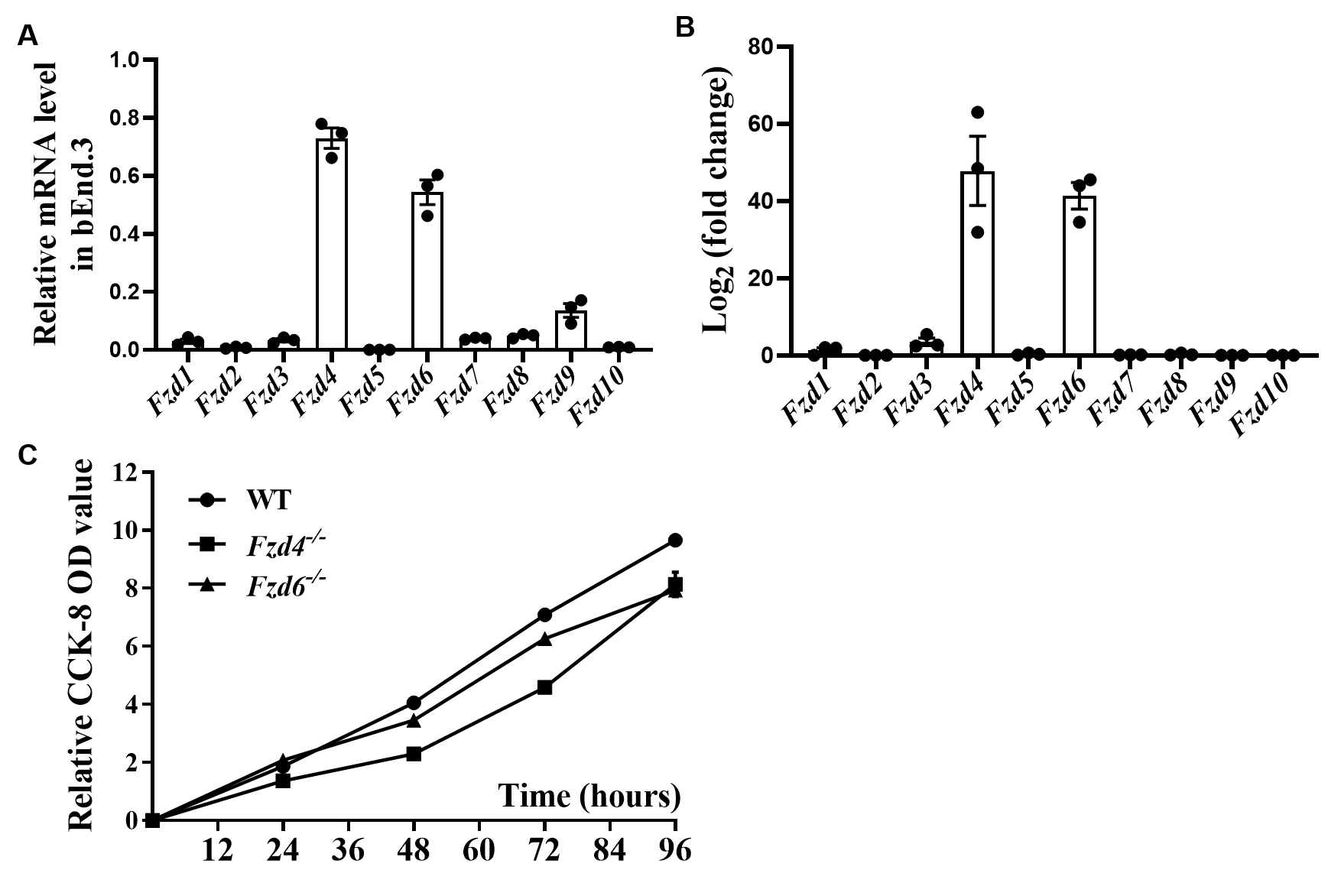

Supplement: Supplementary file 5 — Figure S5 [file CNS-27-1085-s007.tif]

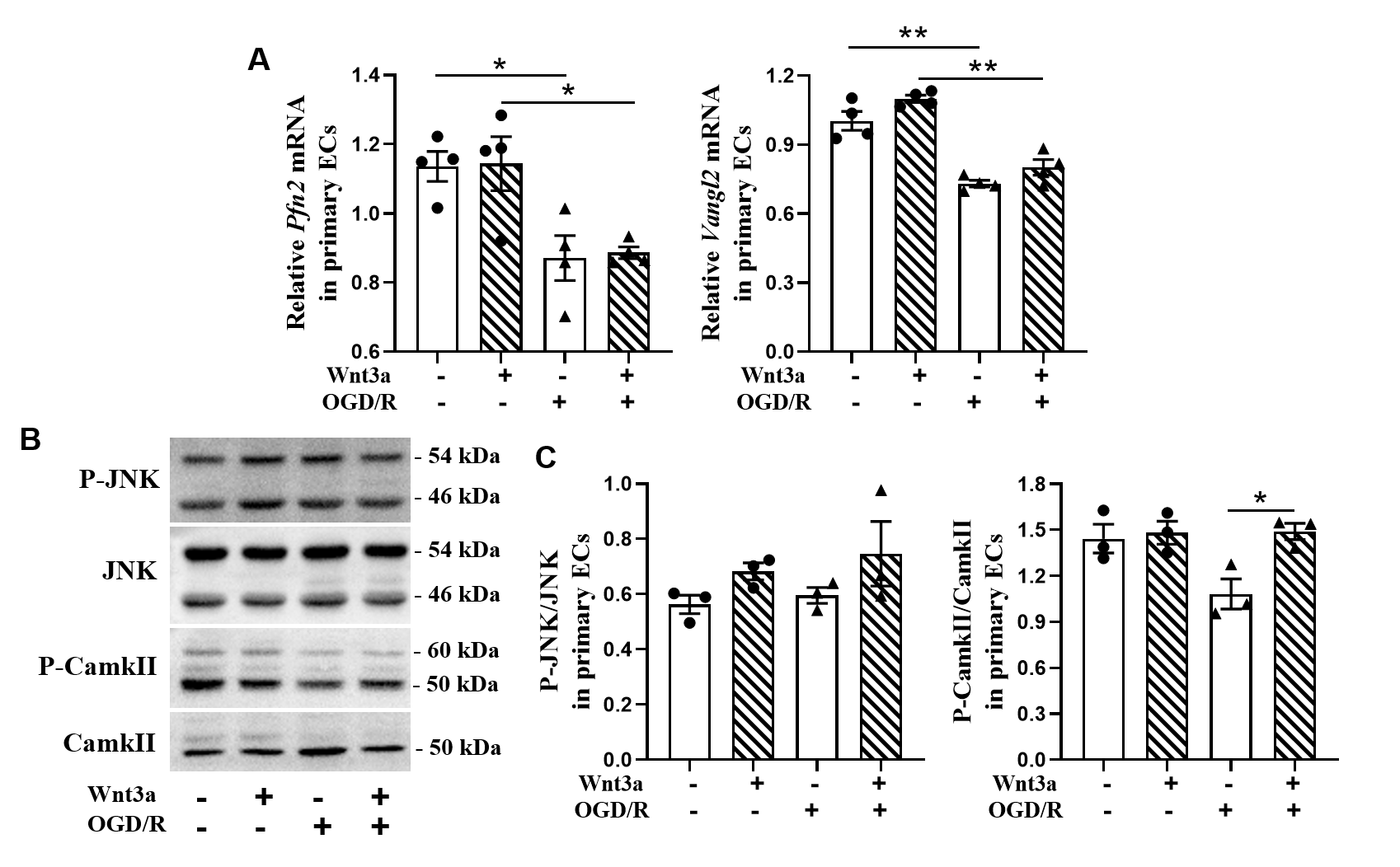

Supplement: Supplementary file 6 — Figure S6 [file CNS-27-1085-s002.tif]
