# Supplementary material for: Normalization of non‐canonical Wnt signalings does not compromise blood‐brain barrier protection conferred by upregulating endothelial Wnt/β‐catenin signaling following ischemic stroke
Source: CNS Neurosci Ther. 2021 May 31;27(9):1085–96. doi: 10.1111/cns.13661 (PMC8339534; doi:10.1111/cns.13661)
Supplement: Supplementary file 7 — Supinfo [file CNS-27-1085-s006.docx]

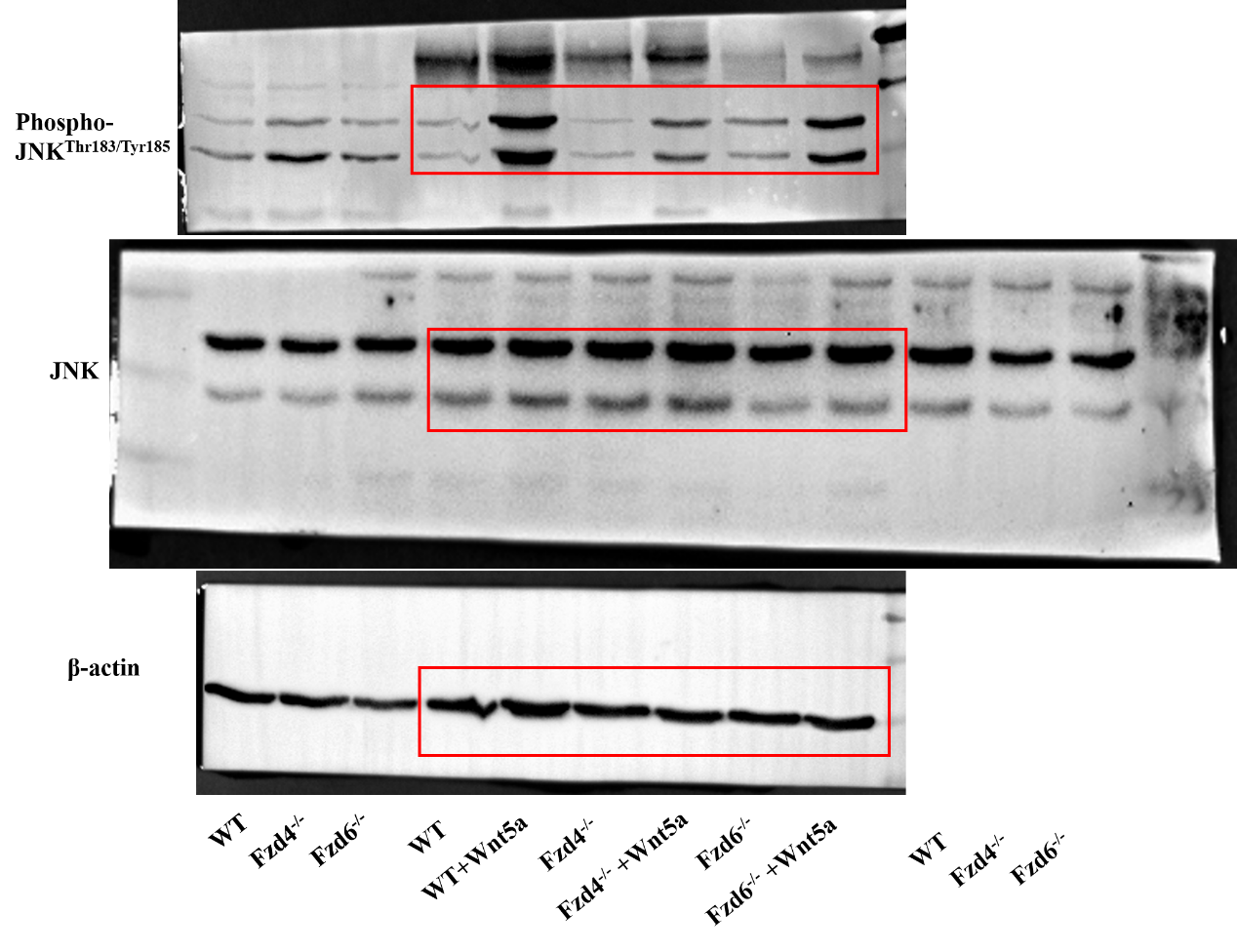


**The original western blot image of Figure 1C**


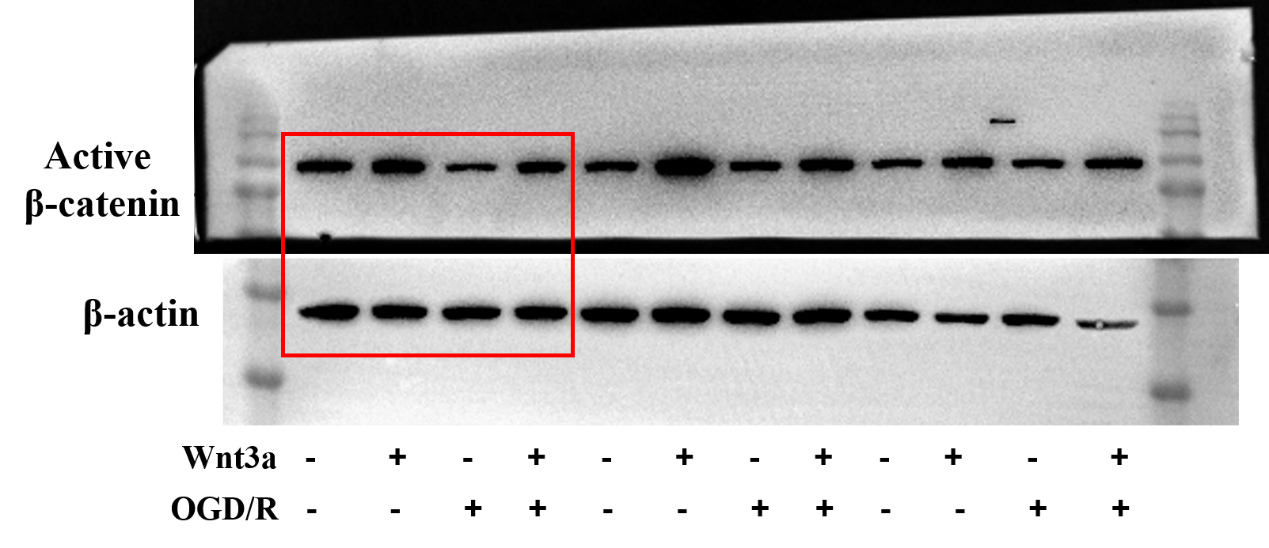


**The original western blot image of Figure 2A**


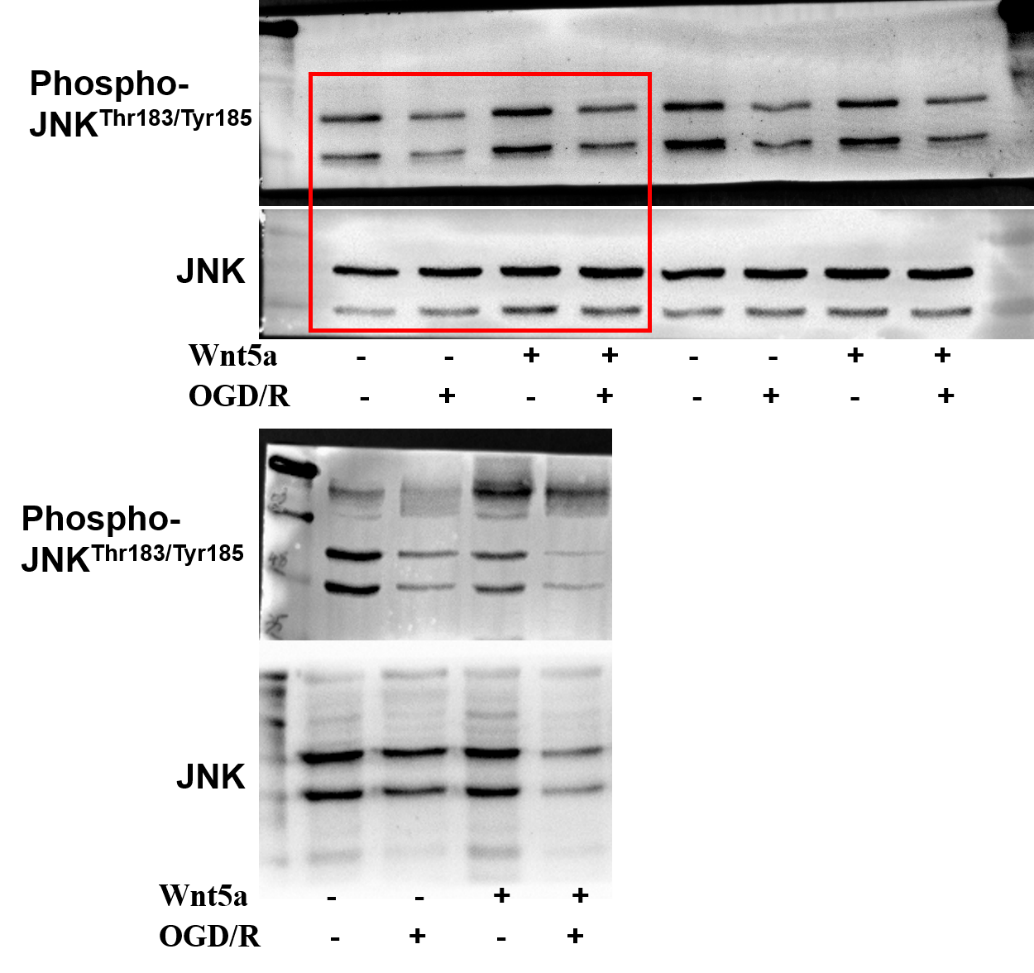


**The original western blot image of Figure 2A**


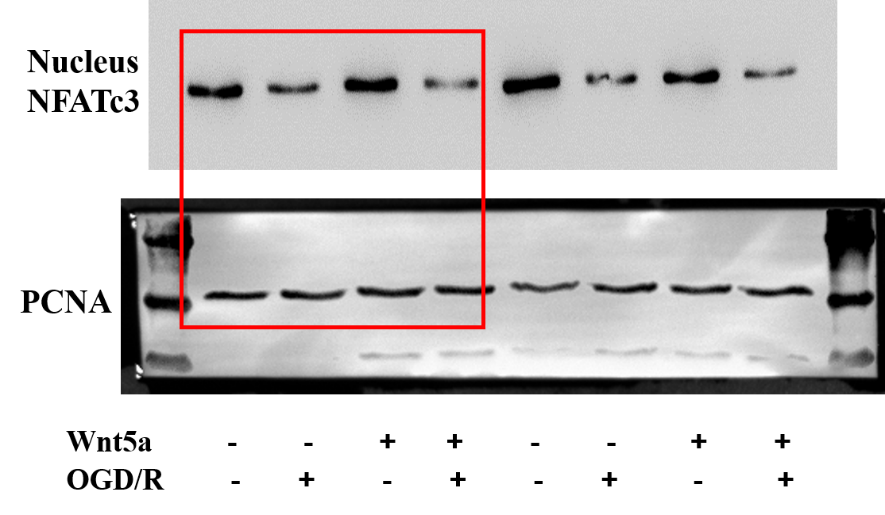


**The original western blot image of Figure 2A**


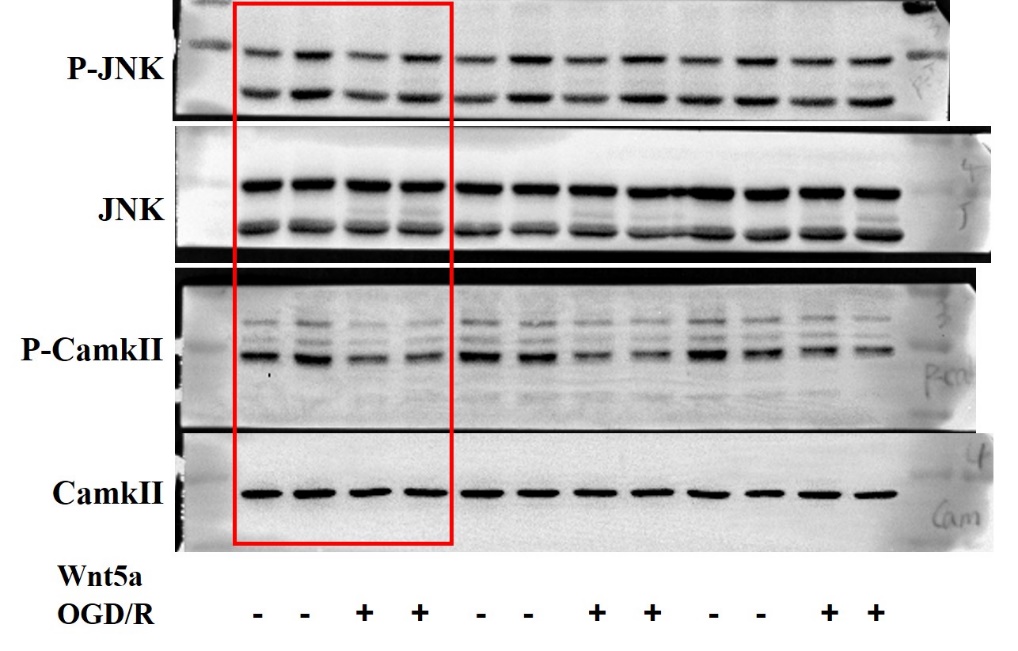


**The original western blot image of Figure 2E**


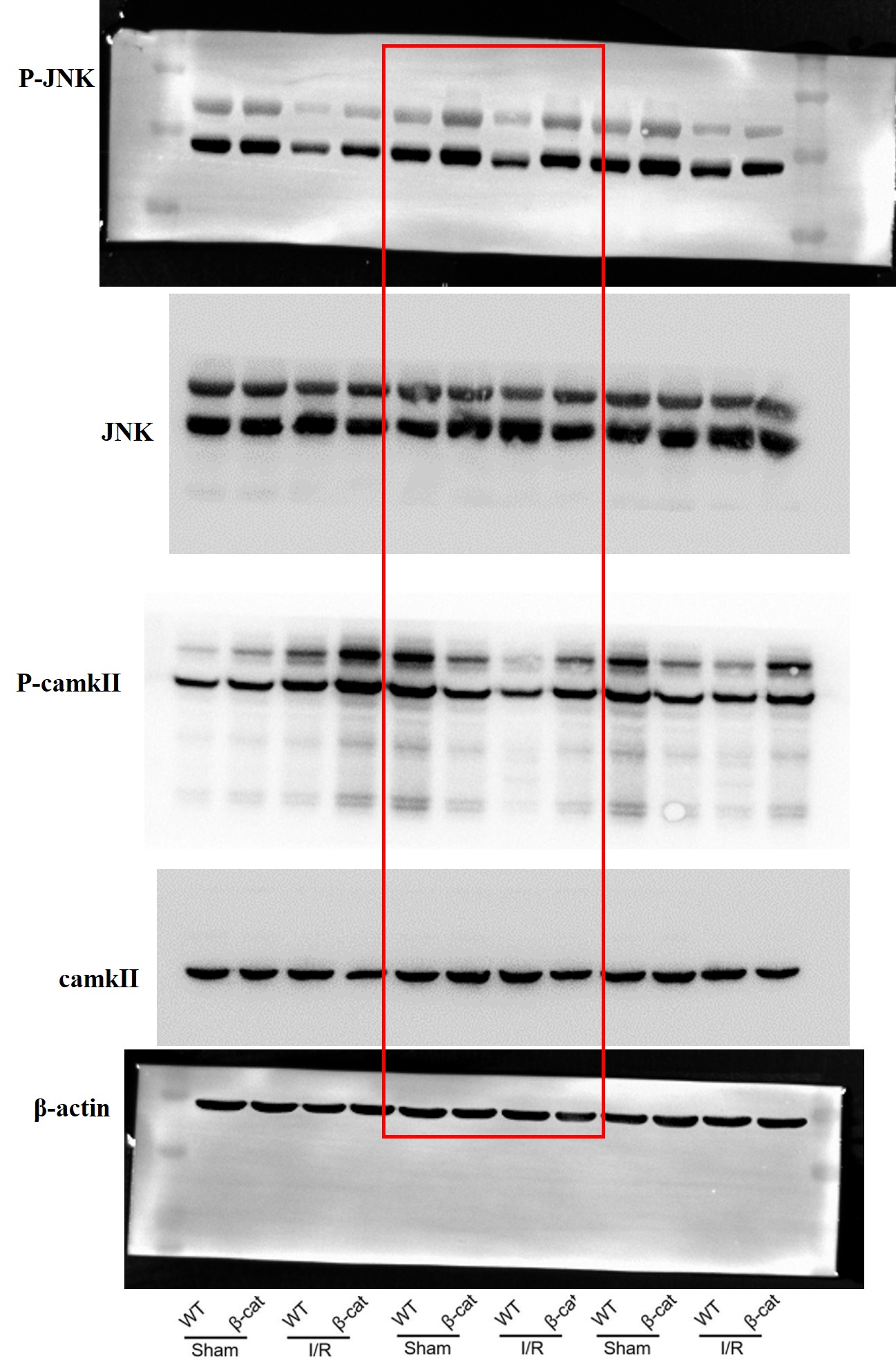


**The original western blot image of Figure 3B**


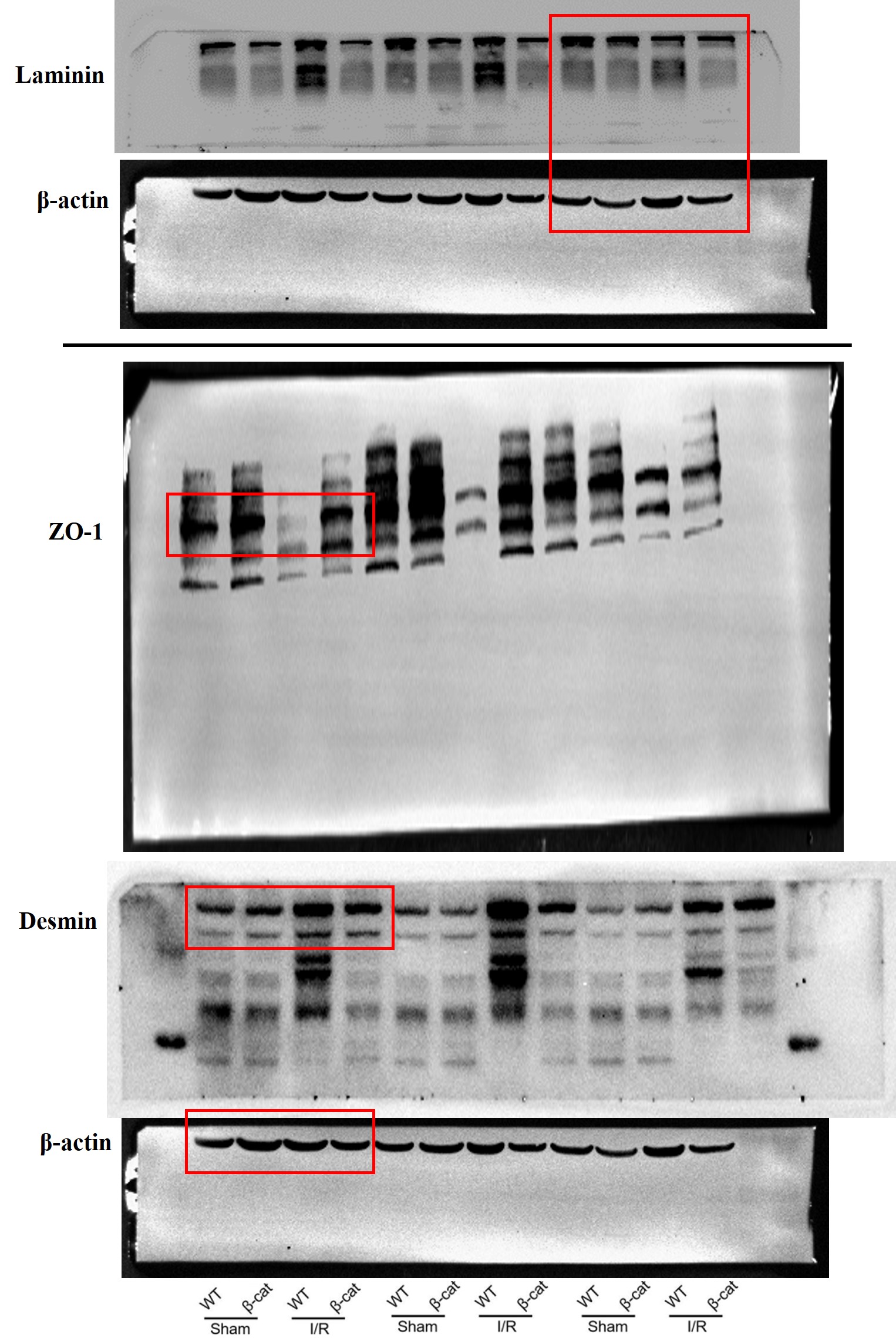


**The original western blot image of Figure 4C**


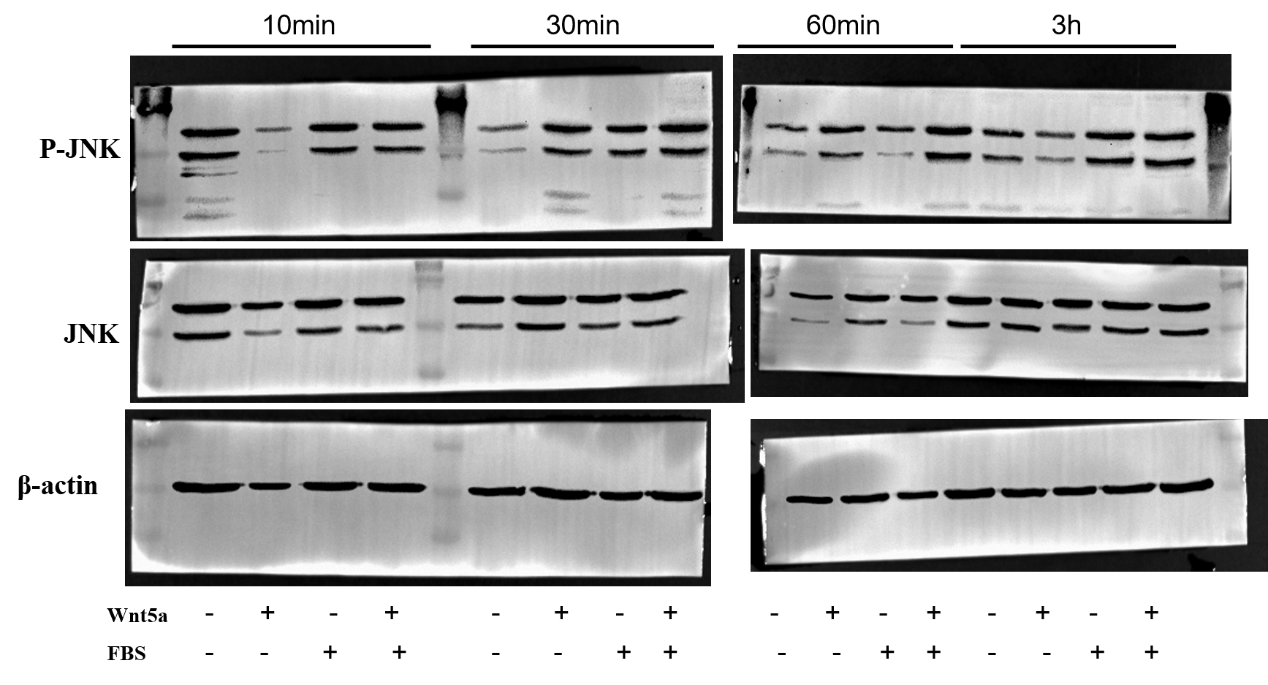


**The original western blot image of Supplementary Figure 2**


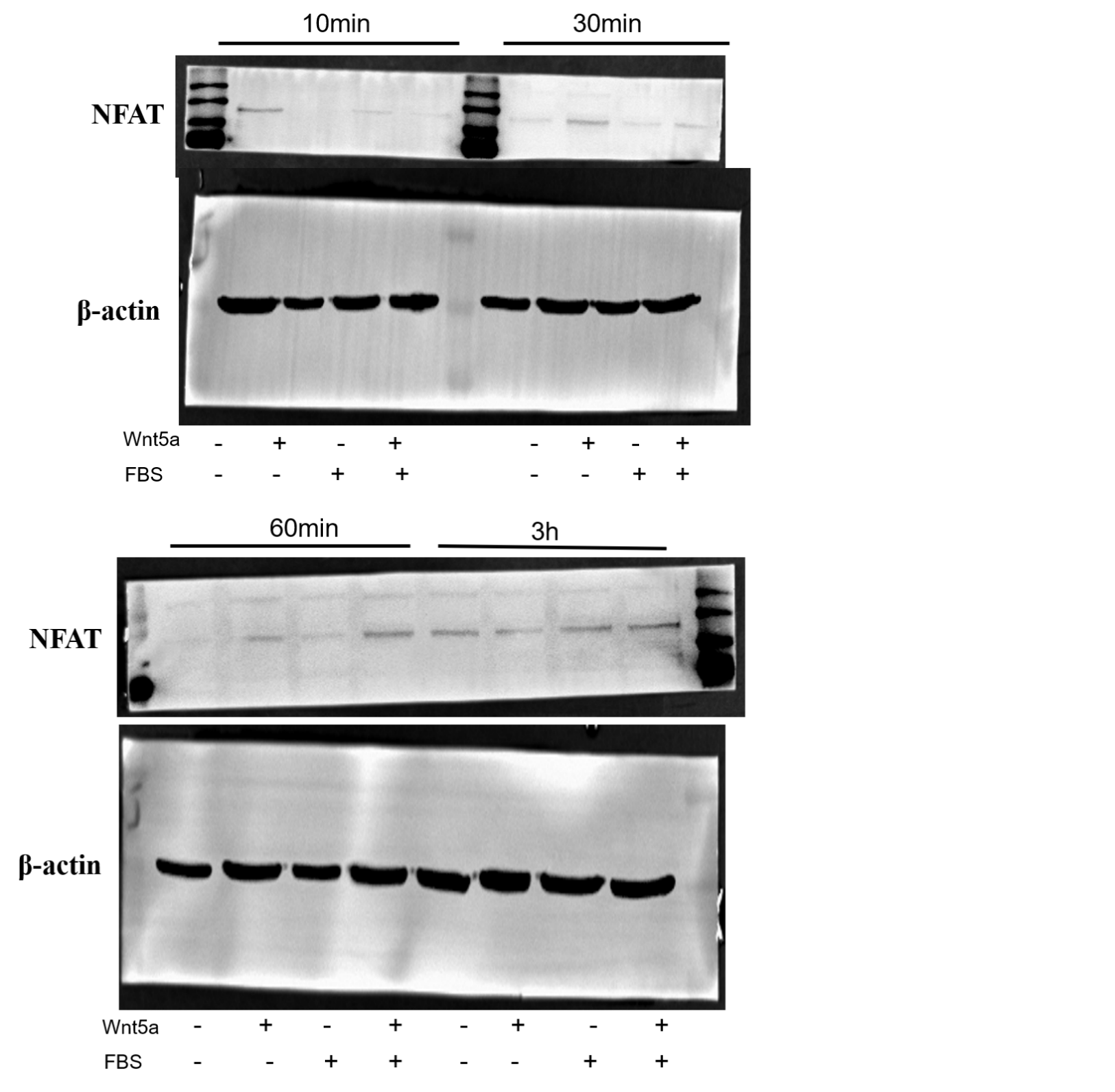


**The original western blot image of Supplementary Figure 2**


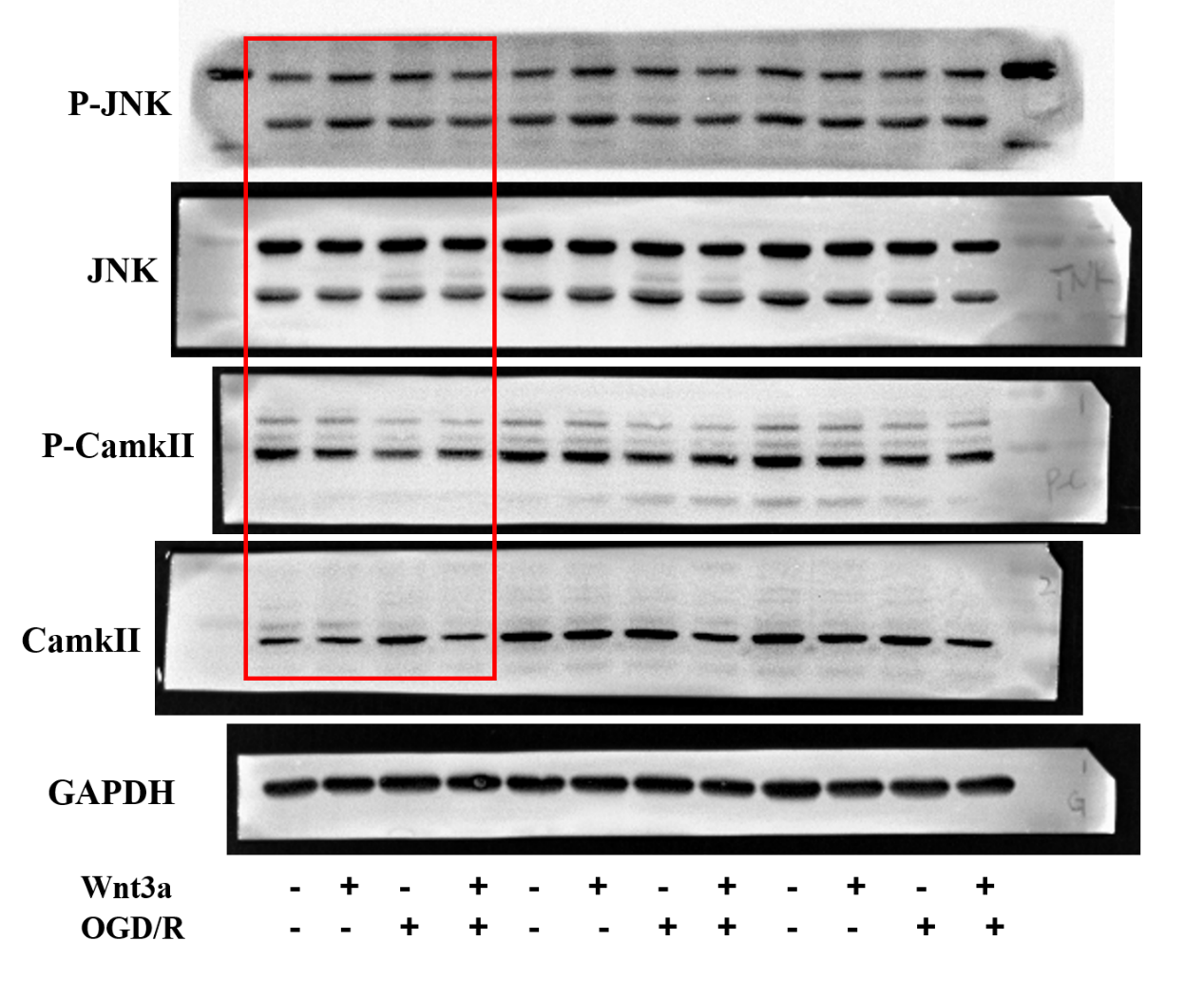


**The original western blot image of Supplementary Figure 6B**
