# Supplementary material for: Normalization of non‐canonical Wnt signalings does not compromise blood‐brain barrier protection conferred by upregulating endothelial Wnt/β‐catenin signaling following ischemic stroke
Source: CNS Neurosci Ther. 2021 May 31;27(9):1085–96. doi: 10.1111/cns.13661 (PMC8339534; doi:10.1111/cns.13661)
Supplement: Supplementary file 8 — Table S1 [file CNS-27-1085-s003.docx]

| **Primer name** | **Sequence (5’ – 3’)** |
| --- | --- |
| Axin2-F | GCCGACCTCAAGTGCAAACTC |
| Axin2-R | GGCTGGTGCAAAGACATAGCC |
| Nkd1-F | AGGAAAGGCATCGAGGAGTG |
| Nkd1-R | TCGCTCAGTCTCTCCATTCTC |
| Apcdd1-F | CGCCTGGAGGGCTTTCAAG |
| Apcdd1-R | GGACCCGACCTTACTTCACAG |
| Pfn2-F | CTACGTGGATAACCTGATGTGC |
| Pfn2-R | TCGCAGTAGCCGACAATGG |
| Vangl2-F | ACTCGGGCTATTCCTACAAGT |
| Vangl2-R | TGATTTATCTCCACGACTCCCAT |
| CamkII-F | ACCGACGACTACCAGCTTTTC |
| CamkII-R | GCAGCATATTCCTGCGTAGATG |
| Fzd1-F | CAGCAGTACAACGGCGAAC |
| Fzd1-R | GTCCTCCTGATTCGTGTGGC |
| Fzd2-F | GCCGTCCTATCTCAGCTATAAGT |
| Fzd2-R | TCTCCTCTTGCGAGAAGAACATA |
| Fzd3-F | ATGGCTGTGAGCTGGATTGTC |
| Fzd3-R | GGCACATCCTCAAGGTTATAGGT |
| Fzd4-F | TGCCAGAACCTCGGCTACA |
| Fzd4-R | ATGAGCGGCGTGAAAGTTGT |
| Fzd5-F | CAACCACGCCAAACCGAATAC |
| Fzd5-R | CAGTCCAGATCCCGAGTGATG |
| Fzd6-F | TCTGCCCCTCGTAAGAGGAC |
| Fzd6-R | GGGAAGAACGTCATGTTGTAAGT |
| Fzd7-F | GCCACACGAACCAAGAGGAC |
| Fzd7-R | CGGGTGCGTACATAGAGCATAA |
| Fzd8-F | ATGGAGTGGGGTTACCTGTTG |
| Fzd8-R | CACCGTGATCTCTTGGCAC |
| Fzd9-F | TTGCTCTATTATTTCGGGATGGC |
| Fzd9-R | CAGGACCACGATAGTTTTGAGTG |
| Fzd10-F | GCAAGCTCCCCAACAAGAAC |
| Fzd10-R | CCCGTCCTTTAGTGGGTGC |

**Supplementary Table 1** The primers of genes used for RT-qPCR. Fzd, frizzled; RT-qPCR, real-time quantitative PCR.
